# Circadian glucocorticoid oscillations preserve a population of adult hippocampal neural stem cells in the aging brain

**DOI:** 10.1038/s41380-019-0440-2

**Published:** 2019-06-20

**Authors:** M. Schouten, P. Bielefeld, L. Garcia-Corzo, E. M. J. Passchier, S. Gradari, T. Jungenitz, M. Pons-Espinal, E. Gebara, S. Martín-Suárez, P. J. Lucassen, H. E. De Vries, J. L. Trejo, S. W. Schwarzacher, D. De Pietri Tonelli, N. Toni, H. Mira, J. M. Encinas, C. P. Fitzsimons

**Affiliations:** 1 Neuroscience Collaboration, Swammerdam Institute for Life Sciences, Faculty of Sciences, Amsterdam Neuroscience, University of Amsterdam, Amsterdam, The Netherlands; 2 Department of Molecular Cell Biology and Immunology, VU University Medical Center, Amsterdam Neuroscience, Amsterdam, The Netherlands; 3 Biomedicine Institute of Valencia (IBV), Consejo Superior de Investigaciones Científicas (CSIC), Valencia, Spain; 4 Cajal Institute, Consejo Superior de Investigaciones Científicas (CSIC), Madrid, Spain; 5 Institute of Clinical Neuroanatomy, Neuroscience Center, Goethe-University Frankfurt, Frankfurt am Main, Germany; 6 Neurobiology of miRNA Lab, Neuroscience and Brain Technologies Department, Istituto Italiano di Tecnologia, Genoa, Italy; 7 Center for Psychiatric Neuroscience, Department of Psychiatry, Lausanne University Hospital (CHUV), Lausanne, Switzerland; 8 Achucarro Basque Center for Neuroscience, Leioa, Spain; 9 Ikerbasque, The Basque Foundation for Science, Bilbao, Spain; 10 University of the Basque Country (UPV/EHU), Leioa, Spain

**Keywords:** Stem cells, Cell biology

## Abstract

A decrease in adult hippocampal neurogenesis has been linked to age-related cognitive impairment. However, the mechanisms involved in this age-related reduction remain elusive. Glucocorticoid hormones (GC) are important regulators of neural stem/precursor cells (NSPC) proliferation. GC are released from the adrenal glands in ultradian secretory pulses that generate characteristic circadian oscillations. Here, we investigated the hypothesis that GC oscillations prevent NSPC activation and preserve a quiescent NSPC pool in the aging hippocampus. We found that hippocampal NSPC populations lacking expression of the glucocorticoid receptor (GR) decayed exponentially with age, while GR-positive populations decayed linearly and predominated in the hippocampus from middle age onwards. Importantly, GC oscillations controlled NSPC activation and GR knockdown reactivated NSPC proliferation in aged mice. When modeled in primary hippocampal NSPC cultures, GC oscillations control cell cycle progression and induce specific genome-wide DNA methylation profiles. GC oscillations induced lasting changes in the methylation state of a group of gene promoters associated with cell cycle regulation and the canonical Wnt signaling pathway. Finally, in a mouse model of accelerated aging, we show that disruption of GC oscillations induces lasting changes in dendritic complexity, spine numbers and morphology of newborn granule neurons. Together, these results indicate that GC oscillations preserve a population of GR-expressing NSPC during aging, preventing their activation possibly by epigenetic programming through methylation of specific gene promoters. Our observations suggest a novel mechanism mediated by GC that controls NSPC proliferation and preserves a dormant NSPC pool, possibly contributing to a neuroplasticity reserve in the aging brain.

## Introduction

Aging imposes an increasing disease burden and the neurological consequences of aging, such as cognitive decline, are particularly deleterious to quality of life [1]. There is substantial heterogeneity in the various changes in brain function associated with aging, suggesting that aging proceeds at different rates due to genetic, environmental, emotional and/or physiopathological factors [2]. Among the latter, alterations in circadian glucocorticoid hormones (GC) rhythms are associated with increased allostatic load and may affect normal aging [3–5]. GC are rhythmically released from the adrenal glands in ultradian near-hourly pulses. These ultradian pulses generate characteristic circadian oscillations in circulating GC levels [6, 7]. GC oscillations develop after the third week of life in mice [8] and induce cyclic glucocorticoid receptor (GR)‐mediated transcriptional regulation, or gene pulsing, in vitro [9] and also in vivo in the hippocampus [10]. Alterations in GC oscillations are observed in aged mammals, including mice [11] and humans [6]. GC oscillations have been implicated in the regulation of cortical plasticity [12], anxiety-like behavior [13], and the diurnal rhythm of neural stem/precursor cells (NSPC) proliferation in the dentate gyrus (DG) [14].

NSPC in the sub-GZ (SGZ) of the DG proliferate and generate new neurons in the adult hippocampus across the lifespan of most mammals [15–21]. Several studies have documented an age-associated decline in NSPC proliferation, suggesting an age-dependent exhaustion of the NSPC pool [19, 22–29]. As adult NSPC proliferation may be limited to a finite number of divisions [27], NSPC quiescence could preserve a NSPC pool that contributes to neuroplasticity reserve and preservation of hippocampus-dependent cognitive functions during aging [19, 30–33]. However, this hypothesis remains controversial and subject to debate [34–37]. In particular, the underlying molecular mechanisms involved are still unknown and require detailed characterization.

NSPC dynamically and selectively respond to GC, which strongly inhibit NSPC proliferation [23, 38–40]. In mice, GC acting through the GR have direct effects on NSPC differentiation and functional integration within hippocampal circuits [41]. In old rats, adrenalectomy (ADX) increases NSPC proliferation in the hippocampus, whereas lifelong GC reduction increases AHN and prevents age-related memory disorders [23, 39, 42]. Interestingly, ADX induces a cellular phenotype in the DG that is very similar to the one induced by GR knockdown, i.e., a significant increase in the number of DCX+ cells and immature neurons with an ectopic location and multiple primary dendrites, indicating that the GR is of critical importance in the regulation of newborn neuron maturation [41]. However, ADX is a surgical strategy that will affect all GC-responsive cell types and remove several other adrenal hormones as well, making the identification of a direct link to cell-type specific effects impossible. The effects of GC on adult hippocampal neurogenesis (AHN) are age-dependent, as life-long GC suppression from early life onwards does not enhance AHN [43]. Therefore, the relationship between GC, NSPC proliferation and AHN is complex and remains incompletely characterized. Importantly, in young adult mice, NSPC populations exhibit differences in GR expression and response to GC stimulation [41, 44, 45].

Here, we show for the first time that GC oscillations are associated with the preservation of GR-expressing NSPC populations in the aging DG, suggesting a novel mechanism that controls the maintenance of NSPC in the aging brain and presenting a possible source of neuroplasticity reserve that could be exploited to sustain hippocampus-dependent cognitive functions throughout life.

## Results

### GR^+^ NSPC populations persist into old age and decay with different kinetics in vivo

NSPC were classified based on the expression of Nestin-GFP and GFAP [16, 46, 47]. Specifically, Nestin-GFP^+^/GFAP^+^ with characteristic radial glia-like morphology were classified as Type-1 cells. Type-2a cells were Nestin-GFP^+^/GFAP^+^, with horizontal morphology and Type-2b cells were Nestin-GFP^+^/GFAP^−^, also with horizontal morphology. Type-1, -2a and -2b cells were observed in animals of all ages (Fig. S1C–I). The numbers of proliferative NSPC decreased with age in Nestin-GFP mice [27] (Fig. S1 A, B). Furthermore, extra-sum-of-squares *F*-testing for best-fit decay curves showed that the total Nestin-GFP^+^ NSPC population decayed exponentially during aging (Fig. S1J). Importantly Nestin-GFP expression was consistent with native Nestin expression over time and was unaffected by aging in individual Type 1 NSPC [27] (Fig. S2A–C). Interestingly, Type-1, -2a, and -2b cells decayed following different patterns. Type-1 and -2a cells decayed linearly, while Type-2b cells followed exponential decay kinetics (Fig. S1K). The volume of the granule zone (SGZ plus granule cell layer (GCL)) did not change significantly with age (Fig. S1J). These data demonstrate that Type-1 and -2a NSPC persist into old age, while Type-2b cells are depleted earlier following an exponential decay.

**Fig. 1:**
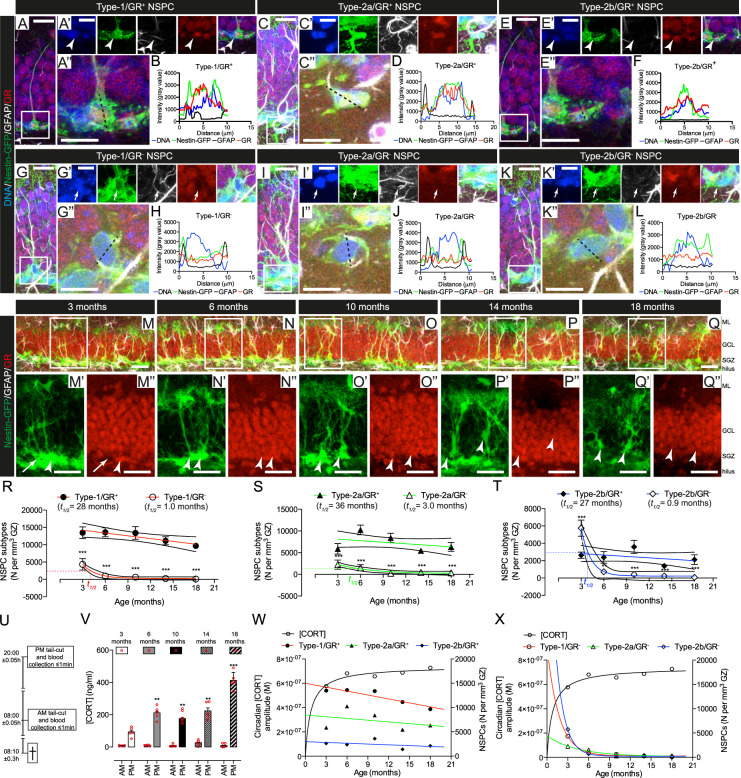
The preservation of NSPC populations is associated with GR expression and age-related changes in the amplitude of circadian CORT oscillations. **a** Representative example of Nestin-GFP^+^/GFAP^+^/GR^+^ NSPC with characteristic vertical process and triangular cell-body in the SGZ of the DG. **a’** The boxed area in A is magnified and channels split and *Z*-stacked, showing the expression of individual markers. Arrowhead: cell soma. **a”** The dashed black line shows a transversal cell section. **b** Histogram of the transversal section in (**a”**), showing fluorescent intensity signals for DNA (blue), GFP (green), GFAP (black) and GR (red). Representative examples of **c**–**d** Type-2a/GR^+^, **e**–**f** Type-2b/GR^+^, **g**–**h** Type-1/GR^−^, **i**–**j** Type-2a/GR^−^, and **k**–**l** Type-2b/GR^−^ NSPC. In all cases cells with intensity value ≥1500 across the nucleus were considered GR^+^ (Fig. S2D). NSPC in the DG of **m** 3, **n** 6, **o** 10, **p** 14, or **q** 18-month-old mice. The boxed areas are shown magnified in the panels below each image. Arrows: Nestin-GFP^+^/GR^−^ Type-1 NSPC; arrowheads: Nestin-GFP^+^/GR^+^ Type-1 NSPC. Scale bars represent 40 μm (**m**–**q**”); 20 μm (**a**, **c**, **e**, **g**, **i**, and **k**); 15 μm (**a’**, **c’**, **e’**, **g’**, **i’**, and **k’**) and 10 μm (**a”**, **c”**, **e”**, **g”**, **i”**, and **k”**). **r** Best-fit curves and 95% confidence intervals of Type-1 GR^+^ (solid circles) GR^−^ (open circles); **s** Type-2a GR^+^ (solid triangles) and GR^−^ (open triangles) or (**t**) Type-2b GR^+^ (solid diamonds) and GR^−^ (open diamonds) cell numbers. Data points indicated by the different shapes are mean ± SEM (*n* = 5 mice, *p < 0.05, **p < 0.01, ***p < 0.001, one-way ANOVA) and NSPC population half-lives (*t*_1/2_) are indicated in the figures. GR^−^ populations fitted exponential decay curves (*p* *<* 0.05, *F*-test, calculated *t*_1/2_ *=* 1.02 (Type-1), 3.0 (Type-2a), and 0.9 months (Type-2b) NSPC, respectively). GR^+^ populations fitted linear decay curves (*p* *<* 0.05, *F*-test, calculated *t*_1/2_ *=* 28 (Type-1), 36 (Type-2a) and 27 months (Type-2b) NSPC, respectively). Best curve fit comparisons are shown in Figure S2F-K. **u** Time-windows of blood collection. **v** AM and PM plasma [CORT] at different ages in mice. Bars are mean ± SEM and red circles individual data points (animals) (*n* = 5 mice, *p < 0.05, **p < 0.01, ***p < 0.001, vs. 3-month-old, one-way ANOVA). Calculated circadian CORT amplitude (black line) vs. **w** GR^+^ or **x** GR^−^ Type-1 (red lines), -2a (green lines) and -2b (blue lines) NSPC numbers at different ages in mice

We next characterized GR expression in Type-1, -2a, and -2b cells in 3- to 18- month-old Nestin-GFP mice (Fig. 1a–q, Fig. S1L, Fig. S2D). The relative abundances of GR^+^ and GR^−^ populations of Type-1, -2a, and -2b cells changed with age (Fig. S1L), in agreement with previous studies showing heterogeneous GR expression in NSPC populations in young animals [41, 44, 48]. At 3 months of age, most Type-1 and -2a cells were GR^+^, whereas the majority of Type-2b cells were GR^-^ at this age. However, from 6 months of age on, GR^+^ cells predominated in all NSPC populations. This predominance of GR^+^ NSPC populations persisted throughout middle and into old age (Fig. S1L). Thus, a marked depletion of GR^−^ NSPC takes place in DG earlier than anticipated from previous studies [44]. Interestingly the decay of GR^−^ NSPC populations fitted best to an exponential decay, while the decay of GR^+^ populations fitted best to a linear model (Fig. 1r–t, Fig. S2F–K).

### The predominance of GR^+^ NSPC populations correlates with an age-associated increase in the amplitude of circadian GC oscillations in vivo

Corticosterone (CORT) concentrations were measured in plasma samples collected at AM (08:00, lights on) and PM (20:00, lights off), representing the nadir and the peak of circadian GC oscillations, respectively (Fig. 1u). CORT AM levels remained stable with age, while PM peak levels were increased in all age groups compared to 3-month-old mice (Fig. 1v), indicating an age-associated increase in the amplitude of circadian GC oscillations that correlated negatively with the numbers of GR^−^ NSPC (Fig. 1w, x and Fig. S3).

### Disruption of circadian GC oscillations in young mice induces NSPC to enter a reversible non-proliferative cellular state in vivo

**Fig. 2:**
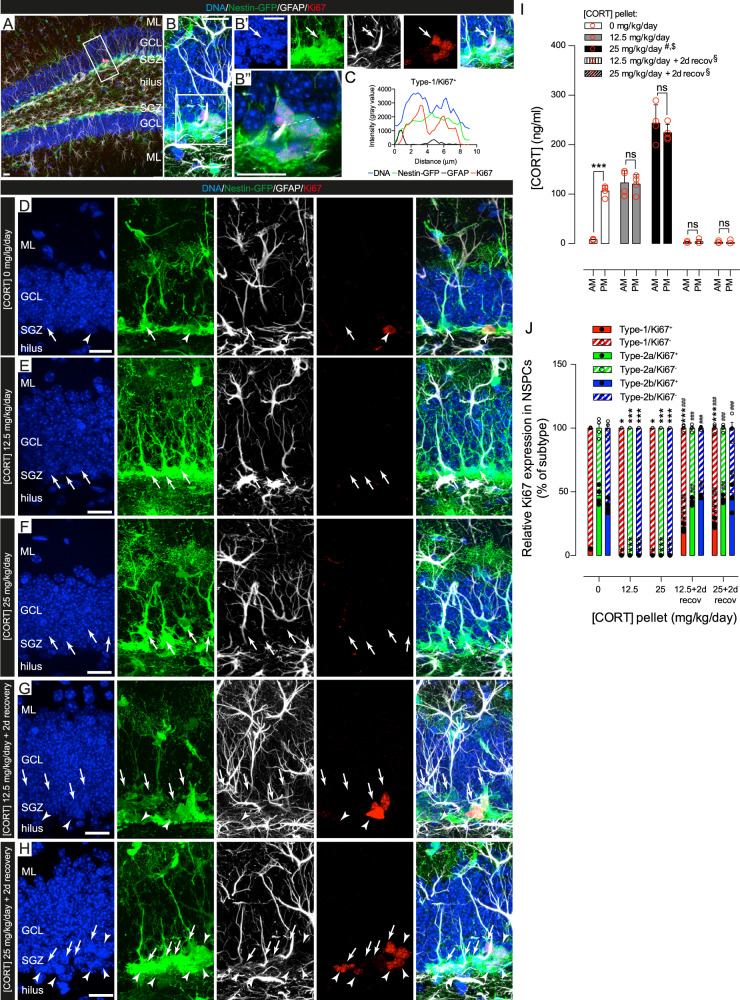
Disruption of GC oscillations in 3-month-old Nestin-GFP mice induces reversible NSPC quiescence. **a** Representative example of Nestin-GFP (green), GFAP (white) and Ki67 (red) immunoreactivity in the DG of 3-month-old Nestin-GFP mice. The boxed area shows a cluster of Nestin-GFP^+^/GFAP^+^/Ki67^+^ NSPC. **b** Magnification of area boxed in (**a**). **b’** Same area further magnified with channels split and *Z*-stacked, showing the expression of individual markers. Arrow: cell soma. **b”** The dashed white line shows a transversal cell section. **c** Histogram of the transversal section in (**a”**), showing fluorescent intensity signals for DNA (blue), GFP (green), GFAP (black), and Ki67 (red). Representative *Z*-stacked confocal images of NSPC in the DG of mice treated for 7 days with **d** 0, **e** 12.5, **f** 25 mg/kg/day [CORT] pellets or allowed to recover for 2 days after removal of a **g** 12.5 and **h** 25 mg/kg/day [CORT] pellet. Arrows: Type-1 cells; arrowheads: Type-2a/2b cells. Scale bars represent 20 μm (**a**, **b** and **d**, **h**), 15 μm (**b’**) and 10 μm (**b”**). **i** AM and PM plasma CORT levels after the treatments indicated in the graph legends. Bars are mean ± SEM [CORT] and red circles individual data-points (animals). Statistical comparisons were done using one-way analysis of variance test with Tukey’s post hoc test for multiple comparisons (*n* = 4 mice, ***p *<* 0.001, AM vs. PM in 0 mg/kg/day, ns *p* *>* 0.05, AM vs. PM in both 12.5 and 25 mg/kg/day and after 2 day recovery; ^#^*p* *<* 0.001, 25 mg/kg/day AM and PM vs. 0 mg/kg/day AM and PM, respectively; ^$^*p* *<* 0.05, 25 mg/kg/day AM and PM vs. 12.5 mg/kg/day AM and PM, respectively; ^§^*p* < 0.001, both AM and PM in 12.5 and 25 mg/kg/day vs. 2-day recovery). **j** Percentages of Ki67^+^ (full bars and full circles) or Ki67^−^ (dashed bars and open circles) of Type-1 (red), −2a (green) and −2b (blue) NSPC, 7 days postimplantation with 0, 12.5, 25 mg/kg/day [CORT] pellets and 25 mg/kg/day [CORT] +2-day recovery. Ki67^+^ NSPC were not observed in animals treated with [CORT] 12.5 and 25 mg/kg/day. Bars are mean ± SEM and circles individual data points (animals) (*n* = 4 mice, *p < 0.05, ***p < 0.001, vs. 0 mg/kg/day, one-way ANOVA or ^###^*p* < 0.001, 7-day treatment vs. 7-day treatment +2-day recovery with the same [CORT], one-way ANOVA). Further information in Fig. S4A. ML molecular layer, SGZ subgranular zone, GCL granule cell layer

One-week-long subcutaneous implantation of CORT pellets suppressed GC oscillations and proliferation in the mouse DG (Fig. 2 and Fig. S4A), in agreement with previous reports [49]. We observed that low-dose CORT pellets (12.5 mg/kg/day) were able to fix blood [CORT] to PM peak levels, while high-dose pellets (25 mg/kg/day) induced supra-physiological blood [CORT] (Fig. 2i). Ki67^+^ Type-1, -2a, and -2b NSPC populations were detected in 3-month-old mice with oscillating GC levels, but were not observed in mice of the same age implanted with CORT pellets (12.5 and 25 mg/kg/day) (Fig. 2j). Cell proliferation was reinstated in all NSPC populations 2 days after removal (2-day recovery, Fig. 2j) of the CORT implant and was significantly increased in Type-1 cells as compared to vehicle control groups (Fig. 2j). As the implantation of high-dose CORT pellets (25 mg/kg/day) did not result in stronger inhibition of NSPC proliferation as compared to low-dose ones (12.5 mg/kg/day) (Fig. 2j), low-dose pellets (12.5 mg/kg/day) were used in the rest of the experiments. These data indicate a dynamic proliferative response of Type-1 NSPC to the disruption of GC oscillations.

### GR reduction in old mice reactivates proliferation of Type-1 NSPC in vivo

**Fig. 3:**
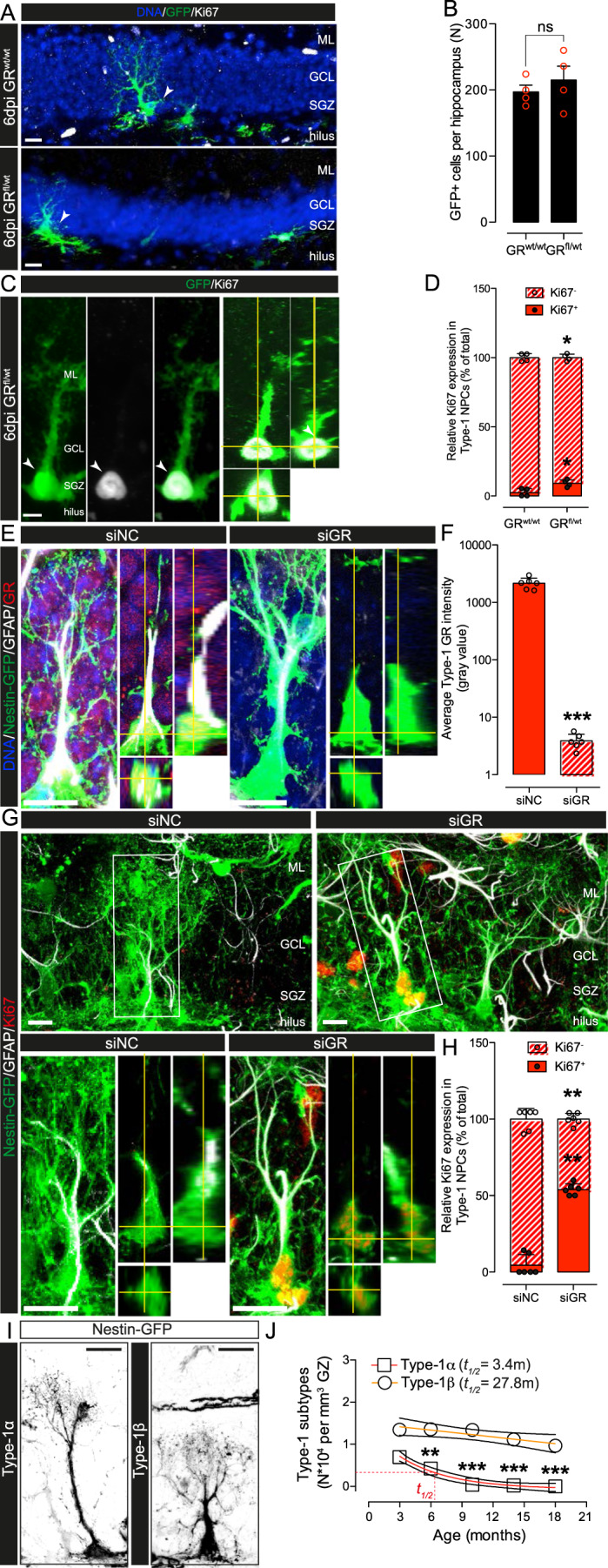
GR knockdown in 12 and 20-month-old mice recovers Type-1 NSPC proliferation. **a** Representative confocal images of GFP^+^ radial glial-like Type-1 NSPC (arrowheads) in 12-month-old (top) GR^wt/wt^ and (bottom) GR^fl/wt^ mice 6 dpi with split-Cre lentiviruses (further details in Fig. S4B). **b** Numbers of GFP^+^ cells per hippocampus in GR^wt/wt^ and GR^fl/wt^ animals. Bars are mean ± SEM GFP^+^ cells per hippocampus of individual mice (red circles) (*n* = 4 mice, ns *p* > 0.05, GR^wt/wt^ vs. GR^fl/wt^, Student’s *t* test). **c** Representative confocal *Z*-stacked image and orthogonal projection of GFP^+^/Ki67^+^ cells with a radial glial-like morphology (arrowheads) in GR^fl/wt^ mice. **d** Relative numbers of Ki67^+^ (full bars and full circles) or Ki67^−^ (dashed bars and open circles) Type-1 cells 6dpi with lentiviruses in GR^wt/wt^ and GR^fl/wt^ animals. Bars are mean) ± SEM and circles individual mice (*n* = 4 mice, *p < 0.05, GR^wt/wt^ vs. GR^fl/wt^, one-way ANOVA). **e** Representative confocal *Z*-stacked image and orthogonal projections of Nestin-GFP^+^/GFAP^+^/GR^+^ Type-1 NSPC 3 dpi with GR (siGR) or negative control (siNC) siRNAs (further details in Fig. S4C–H). **f** GR expression in Type-1 cells 3 dpi with siNC (full bar and open circles) or siGR (dashed bar and open circles). Bars are mean ± SEM GR intensity (gray value) and circles individual mice (*n* = 6 mice, ***p < 0.001, siNC vs. siGR, Student’s *t* test). **g** Top: Nestin-GFP (green), Ki67 (red) and GFAP (white) immunoreactivity in Type-1 cells 3 dpi with siNC or siGR. Bottom: higher magnifications and orthogonal projections of the areas boxed in the top panels. **h** Relative numbers of Type-1 Ki67^+^ (full bars and full circles) or Ki67^−^ (dashed bars and open circles) cells 3 dpi of siNC or siGR. Bars are mean ± SD and circles individual mouse hemispheres (*n* = 6 mice, **p < 0.01, siNC vs. siGR, one-way ANOVA). **i** Representative examples of Nestin-GFP^+^ Type-1α and Type-1β radial glia-like cells found in 3-month-old mice. **j** Best-fit curves and 95% confidence intervals of Type-1α (squares) and Type-1β (circles) numbers vs. age in mice. Data points are mean ± SEM of five mice (*n* = 5, **p < 0.01, ***p < 0.001, vs. 3-month-old, one-way ANOVA). Type-1α and -1β cells fitted best to exponential or linear decay curves, respectively (*p* *<* 0.05, *F*-test, calculated *t*_1/2_ = 3.4 and 27.8 months, Type-1α and -1β, respectively). Scale bars = 15 μm (**a**, **f**, **h**, **j**). ML molecular layer, SGZ subgranular zone, GCL granule cell layer

To characterize the role of the GR on Type-1 cell proliferation in old mice, we used two separate experimental approaches to reduce GR expression. The first approach consisted of a partial genetic inactivation of the GR using a split-Cre system designed for in vivo targeting of Type-1 cells specifically [50] in heterozygous floxed Nr3c1 (GR^fl/wt^) mice [51], (Fig. S4B and “Experimental Procedures”). Secondly, we used a siRNA-mediated reduction of GR expression with previously described siRNAs [41] injected into the DG as described in ref. [52] (Fig. S4C–L). Cells expressing the full Cre-recombinase were visualized using a lentiviral vector expressing a Cre-reporter construct containing a floxed STOP cassette upstream of the enhanced green fluorescent protein (EGFP) gene [53]. Cre-induced recombination in homozygous floxed Nr3c1 (GR^fl/fl^) mice completely abolishes GR expression, while GR expression is only partially reduced in GR^fl/wt^ mice [54], allowing for a better comparison with a siRNA-mediated GR knockdown. Using the split-Cre system (Fig. S4B) we targeted proliferative (Ki67^+^) and nonproliferative (Ki67^−^) Type-1 NSPC in 12-month-old mice (Fig. 3a–c). We found a fourfold increase in proliferative EGFP^+^ Type-1 NSPC in GR^fl/wt^ mice compared to GR^wt/wt^ controls (Fig. 3d). In control experiments using Nestin-GFP mice, we found that GFP+ Type-1 cells readily took up Cy3-labeled siRNAs (Fig. S4I–L) and downregulated GFP expression after injection with siRNAs against GFP (Fig. S4C–H). We subsequently used siRNA injections to reduce GR expression in 20-month-old Nestin-GFP mice. siRNA-mediated GR knockdown resulted in a significant increase in the number of Ki67^+^ Type-1 NSPC, as compared to contralateral control hemispheres injected with negative control non-targeting siRNA (Fig. 3e–h). Type-1 cells present morphological heterogeneity and can be sub-classified into Type-1α cells, that display a long radial process extending into the inner molecular layer, and Type-1β cells, with a short radial process that does not reach the molecular layer (Fig. 3i), which predominate in 8-month-old and older mice [55]. Starting at 10 months of age the vast majority of Type-1 cells we found in the DG were GR^+^ (Fig. 1r, s; Fig. 3j). In 14-month-old and older mice Type-1α cells were practically nonexistent, as described before [27, 55], resulting in a marked predominance of Type-1β cells (Fig. 3j), which were all GR^+^. Reduction of GR expression using genetic (Fig. 3a, c) or siRNA-mediated approaches (Fig. 3e, g) in 12 or 20-month-old mice, respectively, had no apparent effect on Type-1β cell morphology. Overall, these results show that GR reduction in middle-aged and old mice results in Type-1β NSPC reactivation in vivo.

### Primary hippocampal NSPC express the GR and enter a reversible quiescent cellular state after GC treatment in vitro

**Fig. 4:**
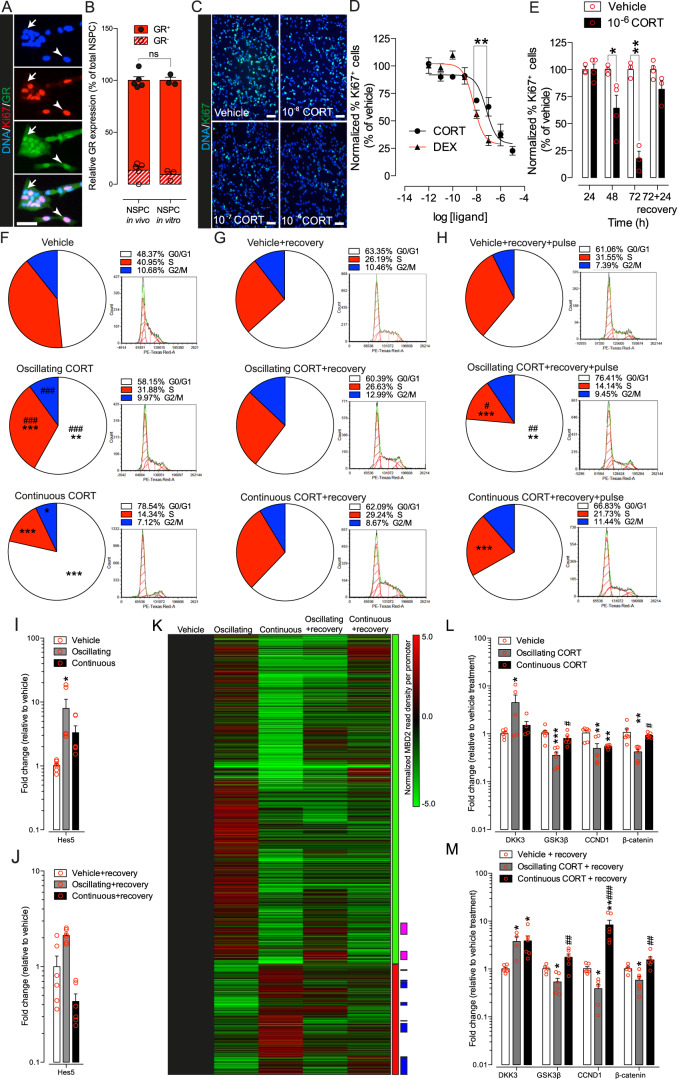
CORT oscillations induce a reversible inhibition of cell proliferation and conserve the responsiveness of NSPC proliferation to CORT exposure in vitro. **a** Nuclear GR^+^/Ki67^+^ (arrowhead) and nuclear GR^−^/Ki67^−^ (arrow) in primary hippocampal NSPC cultures. Nuclei are indicated by the presence of DNA. **b** Relative abundances of GR^+^ (full bars and full circles) and GR^−^ (dashed bars and open circles) NSPC in vivo in 3-month-old Nestin-GFP mice and in vitro NSPC cultures. Bars are relative mean of individual data-points (circles) (% of total NSPC in vivo or in vitro) ± SEM, (*n* = 5 or 3 biological replicates respectively, *p* > 0.05, GR^+^/GR^−^ NSPC in vivo vs. in vitro, one-way ANOVA with Tukey’s post hoc test). **c** Dose-dependent reduction in Ki67^+^ cells (green) in NSPC cultures exposed to CORT or vehicle for 72 h. Cell nuclei (DNA) are shown in blue. Scale bars = 50 μm (**a**, **c**). **d** CORT (black circles) or dexamethasone (DEX; black triangles) dose–response curves. Data are mean normalized proliferative Ki67+ cells (% of vehicle) ± SEM, (*n* = 3 biological replicates, **p < 0.01 on logIC50 of best-fitted curves, *F*-test). **e** Time-dependent effect of 1 × 10^−6^ M CORT on NSPC proliferation (Ki67+ cells), and the effect of a 24 h washout period, (*n* = 3, *p < 0.05 and **p < 0.01 unpaired two-tailed Student’s *t* test). Bars are mean of individual data-points (red circles) ± SEM. Effect on cell proliferation of (**f**) 72 h vehicle, oscillating or continuous 1 × 10^−6^ M CORT; **g** 72 h vehicle, oscillating or continuous 1 × 10^−6^ M CORT followed by a 24 h washout period (recovery) or **h** 72 h vehicle, oscillating or continuous 1 × 10^−6^ M CORT, a 24 h recovery followed by incubation with 1 × 10^−6^ M CORT (pulse). All data are average percentages of total cell populations per cell cycle phase compared with their corresponding vehicle treatment (*n* = 3, *p < 0.05, **p < 0.01 and ***p < 0.001, one-way ANOVA) or continuous vs. oscillating CORT ^#^*p* < 0.05, ^##^*p* < 0.01, and ^###^*p* < 0.001 one-way ANOVA). Changes in multipotency marker Hes5 expression induced by oscillating (gray bars) or continuous CORT (black bars) **i** 72 h or **j** 72 h followed by a 24 h washout period (recovery). Data are mean normalized fold change expression (relative to vehicle) of individual data points (red circles) ± SEM (*n* = 4 biological replicates, *p *<* 0.05 relative to vehicle, one-way ANOVA with Tukey’s post hoc test). **h** Heatmap showing 4767 vehicle normalized differentially methylated gene promoters (72 h of oscillating vs. continuous CORT, MBD2 read density difference ≥ 3). Bars to the right of the heatmap are, green: hypermethylated; red: hypomethylated; pink: stably hypermethylated; blue: stably hypomethylated gene promoter clusters (oscillating vs. continuous CORT, MBD2 read density difference ≥ 3). Changes in DKK3, GSK3β, CCND1, and β-catenin expression induced by oscillating (gray bars) or continuous CORT (black bars) **l** 72 h or **m** 72 h followed by a 24 h washout period (recovery). Data are mean normalized fold change expression (relative to vehicle) of individual data points (red circles) ± SEM (*n* = 4 biological replicates, *p *<* 0.05, **p *<* 0.01, and ***p *<* 0.001 relative to vehicle; ^#^*p* *<* 0.05, ^##^*p* *<* 0.01, and ^###^*p* *<* 0.001 relative to oscillating CORT, one-way ANOVA with Tukey’s post hoc test). All in vitro experiments were run in triplicates and were repeated three times (**n**), unless indicated

Primary hippocampal NSPC cultures have been previously used to model and examine the direct effects of GC on NSPC [41, 56]. In these cultures, as in vivo in 3-month-old Nestin-GFP mice, we found mixed GR^+^ and GR^−^ NSPC populations, with GR^+^ NSPC numerically predominating (Fig. 4a, b). CORT and the specific GR agonist dexamethasone (DEX) reduced the rate of NSPC proliferation as assessed by expression of Ki67 in NSPC cultures, in a dose-dependent manner (Fig. 4c, d). In agreement with their relative affinities for the GR [57], DEX was ~10 times more potent than CORT in its effect on proliferation (IC50 = 5.8 × 10^−9^ M, maximum effect reached at 1 × 10^−7^ M vs. 8.3 × 10^−8^ M, maximum effect reached at 1 × 10^−6^ M, DEX and CORT, respectively). Incubation with both GR agonists resulted in a significant reduction in the number of Ki67^+^ NSPC, leaving ~20% NSPC unaffected (Fig. 4d, e), in agreement with the relative abundance of GR^−^ populations in our NSPC cultures (Fig. 4a, b). The inhibitory effect of CORT was maximal after 72 h of incubation and was reverted 24 h after CORT washout (Fig. 4e). These results indicate that exposure of NSPC to CORT induces a reversible inhibition of NSPC proliferation compatible with cellular quiescence, supporting our observations in vivo (Figs 1–3).

### GC oscillations regulate NSPC cell cycle progression in vitro

We applied a previously described method to model GC oscillations in vitro [9, 58] in which NSPC were treated with pulses (30 min each) of 1 × 10^−6^ M CORT, mimicking the daily CORT peak levels observed in 3-month-old mice (Fig. 1v) or vehicle. To study in more detail the responsiveness of the cell cycle to GC oscillations modeled in NSPC cultures, we applied this pulsatile treatment for intervals of 12 h interspaced with 12 h-long hormone free periods (Fig. S4N, O) for a total of 72 h, a time when the inhibitory effect of CORT on cell proliferation was maximal (Fig. 4e). Cell cycle was analyzed in fixed NSPC using flow cytometry with propidium iodide DNA staining. Oscillatory CORT treatment was compared to continuous stimulation with 1 × 10^−6^ M CORT (Fig. 4f–h, Fig. S4N, O, Fig. S5 and “Experimental Procedures”), as described [9]. Incubation with oscillatory CORT resulted in a significantly smaller percentage of NSPC in the G0/G1 phase of the cell cycle (Fig. 4f), suggesting that CORT oscillations maintain cell cycle entry and progression in NSPC in vitro. Interestingly, the inhibitory effect of continuous CORT incubation on the cell cycle was largely reversed 24 h after CORT washout (recovery, Fig. 4g), in agreement with the transient inhibition of cell proliferation presented in Fig. 4e. Continuous treatment had no significant effects on Hes5 expression, neither after 72 h of treatment nor after 24 h CORT washout (recovery) (Fig. 4i, j). Oscillatory treatment resulted in a transient upregulation of Hes5 72 h after treatment, which disappeared after recovery (Fig. 4i, j), suggesting that the GC treatments did not permanently affect NSPC multipotency, as measured by the expression of Hes5, a marker of multipotent adult NSPC [59]. Overall, these observations in vitro, support the hypothesis that exposure of NSPC to GC oscillations maintain cell cycle entry and proliferation.

Next, we modeled the differences in the amplitude of GC oscillations observed in vivo in young vs. old mice (Fig. 1v) by comparing the effects of oscillatory treatment with 1 × 10^−6^ M CORT (young mice) with oscillatory treatment with 2 × 10^−6^ M CORT (old mice) in vitro. We found that the effects of oscillatory treatment with 2 × 10^−6^ M CORT on the cell cycle in NSPC was indistinguishable from that of oscillatory treatment with 1 × 10^−6^ M CORT (Fig. S5C, D), indicating that GC amplitudes that fully activate the GR result in similar effects on the cell cycle in NSPC. These results suggest that the increased GC amplitude associated with aging in mice after 3 months of age would not result in stronger effects on the cell cycle in NSPC.

We next questioned whether the total daily CORT exposure (TDC), which differed between oscillatory and continuous treatments (Fig. S5A, B), could partially explain the effect of GC oscillations on the cell cycle in NSPC. To approach this question experimentally we incorporated two new GC treatments to our experimental design: nonoscillatory incubation with 0.25 × 10^−6^ M CORT and circadian-only oscillations (12 h on, 12 h off, no ultradian pulses) with 1 × 10^−6^ M CORT. We found that continuous incubation with 0.25 × 10^−6^ M CORT and oscillatory incubation with 1 × 10^−6^ M, which deliver the same TDC calculated as the area under the curve [60, 61], but differ in their oscillatory pattern (Fig. S5B), resulted in different effects on the cell cycle of NSPC in vitro (Fig. S5C). Continuous incubation with 0.25 × 10^−6^ M CORT induced a significant increase in the percentage of cells in the G0/G1 phase, and a concomitant decrease in the percentage of cells in the S phase, compared to oscillatory 1 × 10^−6^ M CORT (Fig. S5D). Similarly, circadian-only oscillations with 1 × 10^−6^ M CORT and oscillatory incubation with 2 × 10^−6^ M CORT, which deliver the same TDC, had significantly different effects on the cell cycle in NSPC (Fig. S5B–D). Interestingly, circadian-only oscillations with 1 × 10^−6^ M CORT had different effects on the cell cycle than oscillatory incubation with 1 × 10^−6^ M CORT (Fig. S5C, D). Of note, oscillatory treatment with 1 × 10^−6^ M CORT or 2 × 10^−6^ M CORT had similar effects on the cell cycle in NSPC, even when their TDCs were significantly different (Fig. S5D). Finally, the effects of vehicle treatment and continuous incubation with 1 × 10^−6^ M CORT were significantly different from all the other treatments and the latter had the most profound effects on the cell cycle in NSPC (Fig. S5C, D). Together, these results indicate that circadian and ultradian oscillation have different effects on the cell cycle in NSPC and that the oscillatory CORT pattern, not the TDC, is responsible for these effects.

To further address the relevance of GC oscillations on NSPC proliferation, we compared the effects of oscillatory incubation with 1 × 10^−7^ M and 1 × 10^−6^ M CORT, which represent ~50 and 100% of the maximal effect of CORT on NSPC proliferation (Fig. 4d, f), on the expression of Sgk-1, a serine/threonine kinase involved in the inhibition of NSPC proliferation by GC [56]. As described by Anacker et al. [56], continuous incubation with 1 × 10^−6^ M CORT induced a significant upregulation of Sgk-1 (Fig. S5E), in agreement with its strong effects on the cell cycle (Fig. 4f). In contrast, we found that oscillatory 1 × 10^−6^ M CORT induced a significant downregulation of Sgk-1 in agreement with its weaker effects on the cell cycle (Fig. 4f), while oscillatory 1 × 10^−7^ M CORT failed to downregulate Sgk-1 (Fig. S5E). Overall, these results indicate that full GR activation during ultradian GC oscillations delivers a biological signal to NSPCs that is independent of the TDC.

**Fig. 5:**
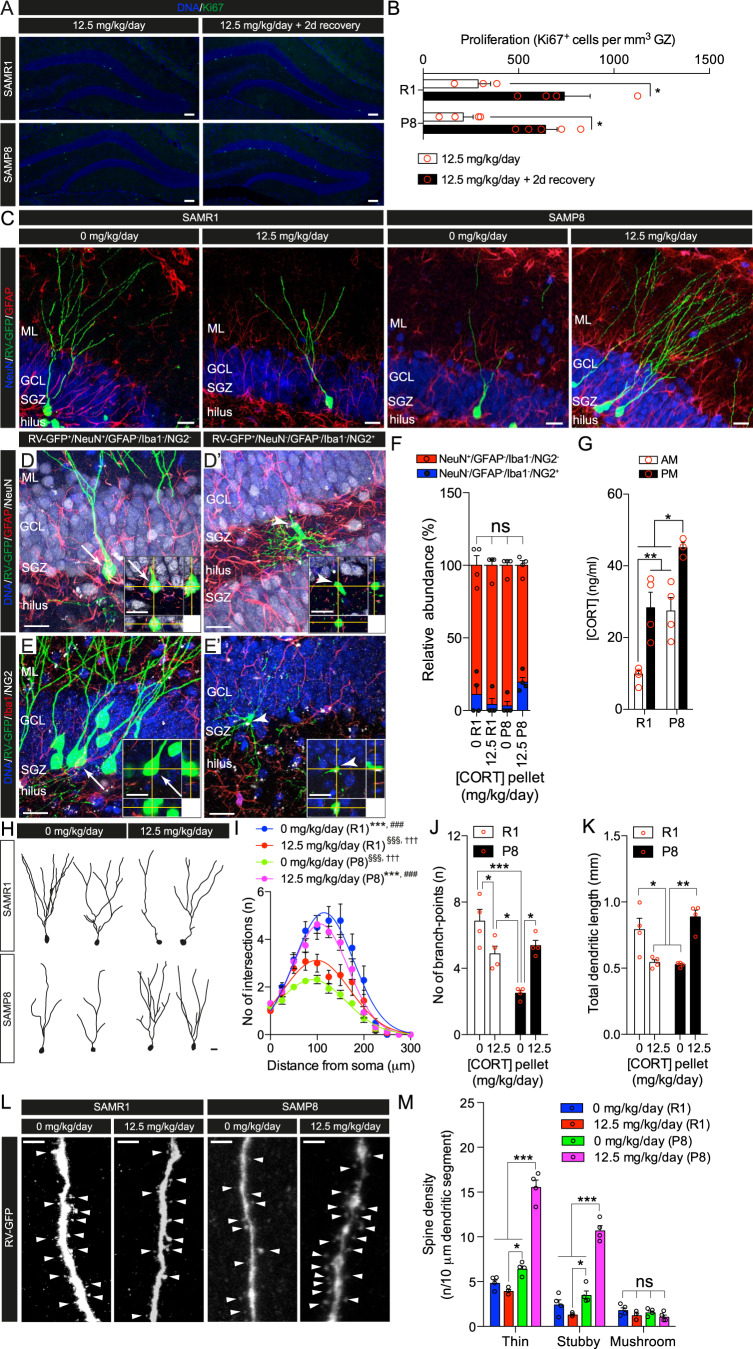
Disruption of GC oscillations in 4-month-old SAMP8 mice induces morphological alterations in NSPC progeny. **a** Representative images of Ki67^+^ cells in the GZ of control senescence-accelerated mouse-resistant 1 (SAMR1) and senescence-accelerated mouse-prone 8 (SAMP8) mice treated for 7 days with 12.5 mg/kg/day CORT pellets and 12.5 mg/kg/day + 2 days recovery (further information in Fig. S4M). **b** Numbers of Ki67^+^ cells in SAMR1 and SAMP8 mice treated with 12.5 mg/kg/day CORT pellets (white bars) or 12.5 mg/kg/day + 2 days recovery (black bars). Bars are mean ± SEM and circles individual mice (*n* = 4 mice, *p < 0.05, 12.5 mg/kg/day vs. 12.5 mg/kg/day + 2 days recovery, two-way ANOVA). **c** Representative *Z*-stacked confocal images of GFP^+^ newborn cells 28 dpi with RV-GFP in the DG of SAMR1 and SAMP8 mice treated for 7 days with 0 or 12.5 mg/kg/day CORT pellets (further information in Fig. S4M). Representative confocal *Z*-stacked images and orthogonal projections (insets) of **d** RV-GFP^+^/NeuN^+^/GFAP^−^ and **e** RV-GFP^+^/Iba1^−^/NG2^−^ cells with neuronal morphology (arrows) or **d’** RV-GFP^+^/NeuN^−^/GFAP^−^ and **e’** RV-GFP^+^/Iba1^−^/NG2^+^ cells with glial morphology (arrowheads). **f** Relative numbers of RV-GFP^+^/NeuN^+^/GFAP^−^/Iba1^−^/NG2^−^ cells with neuronal morphology (red bars and open circles) or GFP^+^/NeuN^−^/GFAP^−^/Iba1^−^/NG2^+^ (blue bars and full circles) cells with glial morphology 28 dpi with RV-GFP in SAMR1 and SAMP8 mice treated with 0 or 12.5 mg/kg/day CORT pellets. Bars are mean ± SEM and circles individual mice (*n* = 4 mice, ns *p* > 0.05, two-way ANOVA). **g** AM (white bars) and PM (black bars) plasma [CORT] in untreated SAMR1 and SAMP8 mice. Bars are mean ± SEM and red circles individual data points (*n* = 5 mice, *p < 0.05, **p < 0.01, two-way ANOVA). **h** Example traces of RV-GFP^+^/NeuN^+^ cells with neuronal morphology (newborn neurons) in SAMR1 and SAMP8 mice treated with 0 or 12.5 mg/kg/day CORT pellets. **i** Sholl analysis of dendritic complexity of GFP^+^/NeuN^+^ cells in SAMR1 and SAMP8 mice treated with 0 mg/kg/day (blue and green line, respectively) or 12.5 mg/kg/day (red and magenta line, respectively) CORT pellets. Data are mean ± SEM (*n* = 4 mice, ***p < 0.001 vs. 12.5 mg/kg/day SAMR1; ^###^*p* < 0.001 vs. 0 mg/kg/day SAMP8; ^§§§^*p* < 0.001 vs. 0 mg/kg/day SAMR1; ^†††^*p* < 0.001 vs. 12.5 mg/kg/day SAMP8 using two-way ANOVA). **j** Number of branching points and **k** average total dendritic length of GFP^+^/NeuN^+^ cells in SAMR1 and SAMP8 animals treated with 0 and 12.5 mg/kg/day CORT pellets. Bars are mean ± SEM and red circles individual mice (*n* = 4 mice, *p < 0.05, **p < 0.01, ***p < 0.001, two-way ANOVA). **l** Representative confocal *Z*-stacked images of GFP^+^ (secondary/tertiary) dendritic segments showing dendritic spines (arrowheads) in SAMR1 and SAMP8 animals treated with 0 or 12.5 mg/kg/day CORT pellets. **m** Spine density per 10 μm dendritic segment in GFP^+^ secondary or tertiary dendrites in SAMR1 and SAMP8 animals treated with 0 mg/kg/day (blue bars and open circles and green bars and open circles, respectively) and 12.5 mg/kg/day (red bars and open circles and magenta bars and open circles, respectively) CORT pellets. Spines were classified in three morphological types: thin, stubby and mushroom. Bars are mean ± SEM and circles individual mice (*n* = 4 mice, *p < 0.05, ***p < 0.001, two-way ANOVA). Scale bars = 100 (**a**), 15 (**c**, **d**, **e’**, **h**) and 8 (**l**) μm. ML molecular layer, SGZ subgranular zone, GCL granule cell layer

Next, we asked whether continuous or oscillating CORT incubations have lasting effects on NSPC responsiveness to CORT. To evaluate this possibility, we exposed NSPC cultures to continuous or oscillating CORT for 72 h, removed CORT from the culture medium for 18 h and then reinitiated CORT treatment (Fig. S4N). This design was based on a population doubling time of 17.8 ± 0.1 h in our NSPC cultures, similar to previous observations in vivo [62], indicating that 18 h after CORT removal NSPC cultures are largely composed of daughter cells that have not been directly exposed to CORT. NSPC cultures exposed to oscillating or continuous CORT treatment showed comparable levels of cells in GO/G1, S and G2/M phase 18 h after CORT removal (Fig. 4g). Importantly, we did not detect significant levels of cells with single-cell DNA content <2N, thus excluding a potential contribution of apoptosis in the conditions tested (Fig. 5f–h). However, daughter cells reacted differentially to incubation with 1 × 10^−6^  M CORT (Fig. 4h). Cells derived from NSPC initially exposed to oscillatory CORT showed a significantly larger proportion of cells in the G0/G1 phase, in comparison to cells derived from NSPC exposed to continuous CORT. This change was compensated by a decrease in the proportion of cells in the S phase (Fig. 4h). These results indicate that cells derived from NSPC initially exposed to oscillating CORT remained sensitive to CORT-induced cell cycle exit, supporting the hypothesis that GC oscillations control NSPC proliferation.

### GC oscillations regulate the expression of DNA methyltransferases (DNMT) in NSPC cultures

DNA methylation at cytosines (5-mC) plays an important role in the regulation of hippocampal NSPC proliferation and survival, potentially providing a basis for long-lasting epigenetic modulation of cellular functions [63, 64]. Interestingly, increased diurnal GC levels are associated with changes in 5-mC and reduced hippocampal volume, indicating that alterations in 5-mC may link hypothalamic–pituitary–adrenal axis dysregulation with structural changes in the hippocampus [65]. Using immunohistochemistry, we found a reduction in 5-mC expression levels in Type-1 cells in 18-month-old mice (Figs S6A and S2E), in which the amplitude of GC oscillations is maximal (Fig. 1v). This observation suggested that GC oscillations may control DNA methylation in NSPC. Previous observations indicate that continuous incubation with DEX downregulates the expression of DNMT-1, -3a and -3, which catalyze and maintain 5-mC, in cultured cortical embryonic NSPC [66, 67]. We exposed NSPC cultures to oscillatory CORT treatment for 72 h in vitro and investigated its effects on DNMTs expression using quantitative polymerase chain reaction (qPCR). Expression of the three DNMTs was downregulated by both oscillatory and continuous CORT treatments (Fig. S6B). However, 24 h after CORT removal, DNMT expression levels remained downregulated in daughter cells derived from NSPC exposed to oscillating CORT, while they were upregulated in daughter cells derived from NSPC exposed to continuous CORT (Fig. S6C). Furthermore, 24 h after washout (recovery) only cells derived from NSPC exposed to continuous CORT reacted to CORT exposure with a significant downregulation of DNMTs (Fig. S6D). These results indicate that GC oscillations maintain a stable DNMT expression profile in daughter NSPC.

### GC oscillations induce global and promoter-specific changes in DNA methylation in NSPC in vitro

Both oscillating and continuous CORT treatments induced significant reductions in global 5-mC as well as in 5-mC levels in specific protein-coding gene promoters, as measured by MBD-isolated Genome Sequencing [68] (Fig. S6E, F). These levels remained significantly reduced 24 h after CORT removal only in cells derived from NSPC exposed to oscillating CORT (Fig. S6E, F). Specifically, unsupervised hierarchical clustering (UHC) analysis of all differentially methylated protein-coding gene promoters between the two CORT regimens (Fig. 4k) revealed that 73% of them were differentially hypermethylated by oscillatory CORT. To identify biological processes that may be regulated by the changes in DNA methylation induced by GC oscillations in NSPC cultures, we performed gene ontology enrichment (GO) analysis of gene sets whose promoters were differentially hypermethylated after exposure to oscillatory CORT (Fig. S6G and Table S1). We found that the most significantly overrepresented biological processes (BPs) were regulation of transcription (6 BPs), metabolic processes, development, differentiation, and phosphorylation. In striking similarity with the BPs overrepresented within hypermethylated gene promoters, GO analysis of the hypomethylated promoters after oscillating CORT (Fig. S6H) identified five BPs linked to regulation of transcription among the most significantly overrepresented. We further identified BPs linked to transport (two BPs), development, phosphorylation, and cell adhesion (Fig. S6H and Table S1). Underscoring the biological relevance of this convergence on BPs, oscillating CORT was associated with hypermethylation of 55 and hypomethylation of 15 gene promoters involved in cell cycle regulation (Table S1). These results indicate a convergence on BP regulating the cell cycle in NSPC. Overall, results in this section indicate that GC oscillations are involved in the control of methylation states in gene promoters associated, among other functions, with cell cycle regulation in NSPC in vitro.

### GC oscillations induce lasting changes in promoter methylation in NSPC

Next, we characterized lasting DNA methylation changes in NSPC derived from cells exposed to oscillating or continuous CORT (Fig. 4k). UHC analysis of promoters differentially methylated in NSPC derived from cells initially exposed to oscillating or continuous CORT, revealed 845 differentially methylated promoters (oscillating vs. continuous treatment) that remained in the same methylation state 24 h after CORT removal (Fig. 4k, pink and blue bars). Further analysis of these stably methylated promoters revealed clusters of stably hypermethylated (214 promoters; Fig. 4k, pink bars) and hypomethylated promoters (631 promoters; Fig. 4k, blue bars). GO analysis of the 214 stably hypermethylated promoters revealed that the most significantly overrepresented BPs were regulation of transcription, cell differentiation and organismal development (Fig. S7A and Table S2). GeneMANIA pathway analysis of the highest overrepresented BPs among the stable hypermethylated promoters revealed a gene network involved in stem cell differentiation (network node: Tbx3; Fig. S7B). Further analysis of overrepresented BPs identified gene networks involved in regulation of glial cell differentiation (network node: Gsx2; Fig. S8A) and regulation of stem cell activation (network node: Ptpn6; Fig. S8B). GO analysis of the 631 stably hypomethylated promoters revealed that the most significantly overrepresented BPs were organismal development, transport, regulation of transcription, and carbohydrate metabolism (Fig. S7C and Table S2). GeneMANIA pathway analysis of the highest overrepresented BPs identified a gene network involved in Wnt signaling (network node: DKK3; Fig. S7D). Further analysis of overrepresented BPs showed gene networks involved in metal-ion transmembrane transport (network node: Slc24a4; Figure S8C) and organic anion transport (network node: Slc7a5; Figure S8D). In agreement with a modulation of Wnt signaling suggested by the lasting promoter hypomethylation and pathway analysis (Fig. S7C, D), we found that the expression of four genes related to the canonical Wnt signaling were differentially affected by CORT treatments (Fig. 4l, m). DKK3 mRNA was upregulated by oscillating CORT, and was unaffected by continuous CORT 72 h after treatment (Fig. 4l), as suggested by its treatment-specific promoter hypomethylation. The expression of GSK3β and β-catenin were downregulated by oscillating CORT and were unaffected by continuous CORT, while CCND1 was downregulated by both treatments in the same time frame (Fig. 4l). Regarding lasting changes, 24 h after washout (recovery), DKK3 remained upregulated (Fig. 4m), whereas GSK3β, CCND1 and β-catenin stayed downregulated in cells derived from NSPC initially exposed to oscillating CORT (Fig. 4m). In contrast, these four genes were upregulated in cells that originated from NSPC treated with continuous CORT (Fig. 4m), suggesting that GC oscillations maintain a stable gene-expression profile of some members of the Wnt signaling pathway in daughter NSPC. Overall, the results in this section imply that GC oscillations, and alterations in them, induce long-lasting changes in NSPC in vitro, which we proceeded to assess in vivo.

### Disruption of GC oscillations in accelerated senescence-prone (SAMP8) mice induces lasting morphological changes in newborn granule neurons in vivo

To assess long-lasting effects of the disruption of GC oscillations on NSPC in vivo in a senescent environment, we used the SAMP8 mouse strain, a senescence-accelerated mouse model with age-related brain dysfunction, in which NSPC proliferation and neurogenesis fall below control levels over time [69–72]. SAMP8 mice present behavioral impairments in object recognition and fear conditioning, compatible with hippocampal dysfunction, and disrupted circadian rhythm as young as 4 months of age, supporting their use as a model of circadian rhythm disturbances associated with pathological aging [73, 74]. Indeed, we found that AM and PM CORT levels were significantly elevated in untreated SAMP8 compared to the genetically related but senescence-resistant SAMR1 mice [75] of the same age (Fig. 5g). We disrupted GC oscillations in 4-month-old SAMP8 and SAMR1 mice using CORT pellet implantation. One week after implantation pellets were removed and after a recovery period of 2 days newborn hippocampal cells were birth-dated using a single injection of a retroviral vector expressing GFP (RV-GFP) [76], and the morphology of newborn neurons was analyzed 28 days after (Fig. S4M). These experimental conditions were consistent with those shown in Fig. 2. SAMP8 and SAMR1 mice were implanted with 12.5 mg/kg/day CORT pellets and let to recover for 2 days, a period of time enough to reinstate proliferation in Nestin-GFP mice (Fig. 2j). As shown in Fig. 5a, b, proliferation measured by the number of Ki67+ cells in the GZ, was significantly higher 2 days after pellet removal in SAMP8 and SAMR1 mice, demonstrating active proliferation at the moment of retrovirus injection. 28 days postinjection (dpi) we found that the majority of the GFP^+^ cells within the GZ were neurons (GFP^+^/NeuN^+^/GFAP^−^/Iba1^−^/NG2^−^ cells with neuronal morphology) (Fig. 5c–f). A small percentage of the GFP+ cells were weakly positive for the proteoglycan NG2 (GFP^+^/NeuN^−^/GFAP^−^/Iba1^−^/NG2^+^ cells) and presented a glial morphology (Fig. 5d–f). Similar cells have been observed before and may represent a distinct class of proliferating glial cells in the DG [77]. The low numbers of these weak NG2^+^ cells were not affected by genotype or treatment (Fig. 5f). GFP^+^ neurons birth-dated with RV-GFP in SAMP8 mice 2 days after CORT pellet removal showed increased dendritic complexity and spine density, with immature spines (thin, stubby) specifically increased, which were seemingly opposite in SAMR1 mice of the same age (Fig. 5h–m). These results indicate lasting changes in dendritic complexity, spine numbers and morphology of newborn granule neurons derived from NSPC exposed to disrupted GC oscillations in SAMP8 mice.

## Discussion

Depletion of NSPC populations and/or the loss of their proliferative capacity have been proposed as causative factors contributing to the age-associated decline in AHN, but the underlying cellular mechanisms remain poorly characterized [27]. Our results indicate that GR^−^ NSPC subpopulations rapidly deplete and GR^+^ subpopulations lose their proliferative capacity with advancing age. GC oscillations, acting through the GR, play a key role in controlling the activation of quiescent NSPC, and thereby may determine the extent of AHN throughout aging.

Our key observations, shown schematically in Fig. S9, are as follows: (1) NSPC populations expressing the GR predominate in the DG starting at middle age and are still present in significant numbers in old mice. (2) The preponderance of GR^+^ NSPC populations is first observed in 6-month-old mice. (3) In older mice, in which the amplitude of GC oscillations is maximal, GR knockdown results in a strong activation of Type-1 cells, which is scarce in control animals of the same age. (4) In vitro, GC oscillations control cell cycle progression, DNMT expression and DNA methylation in specific gene promoters in NSPC. (5) Although some of the changes in promoter methylation were transient, a large number were preserved in daughter NSPC, and affected genes involved in cell processes such as cell cycle control and the canonical Wnt signaling pathway. (6) In a mouse model of accelerated aging, disruption of circadian GC oscillations results in lasting morphological changes in newborn granule neuron morphology, indicating alterations in their connectivity.

Mathematical modeling suggests that the age-associated decrease in AHN is best fit to an exponential decay [24]. Here, we show that GR^−^ Type-1, -2a and -2b NSPC populations had shorter calculated half-lives and decayed exponentially with age, however, their GR^+^ counterparts had significantly longer calculated half-lives and decayed following linear kinetics. These differences in decay kinetics suggest that a population of GR^+^ NSPC is preserved in the aging DG. Previous studies have shown that GR knockdown preferentially in Type-3 (late progenitor) cells had no significant effect on total proliferation in the GCL of young (1.5-month old) mice, although these cells contribute to the pool of proliferative precursor cells [41, 78]. Here, we show that CORT pellet implantation suppresses virtually all proliferation in Type-1 and -2 cells in the GCL of 3-month-old mice where both GR+ and GR− NSPC coexist, in agreement with several previous studies indicating that GC control NSPC proliferation both by direct and indirect mechanisms [56, 79, 80]. In NSPC cultures, where the direct effects of GC can be more easily interpreted [56], CORT and the specific GR agonist DEX inhibited NSPC proliferation, leaving a 20% of cells unaffected, in agreement with the relative abundance of GR-NSPC in the cultures.

Previous studies have indicated that the amplitude of GC oscillations is a key determinant of GC’s biological actions and that these oscillations cyclically activate the GR in the hippocampus [7, 10, 58]. We found that the amplitude of GC oscillations increased with age in mice, reaching a plateau before middle age. This finding is in agreement with previous observations in rats and mice, in which changes in circadian GC oscillations were interpreted as age-associated adaptations of HPA-activity and adrenal sensitivity to ACTH [11, 81]. Albeit CORT levels were not measured in 6-month-old mice, Dalm et al. [11] found an increased circadian amplitude in 9-month-old mice compared to 3-month-old mice. Here, we report that the increase in the amplitude of GC oscillations correlated with a rapid disappearance of GR^-^ NSPC populations. Consistent with these observations, disruption of GC oscillations in vivo in young mice induced a strong inhibition of NSPC proliferation, which was reinstated after CORT pellet removal. In particular, Type-1 cells emerged from this CORT-induced transient inhibition of cell proliferation with a significantly increased proliferation rate. Our findings are consistent with previous studies in young rats, in which treatment with DEX inhibited proliferation in the DG and systemic treatment with a GR antagonist reverted the inhibition of AHN induced by stress [82, 83]. However, our experiments in vitro demonstrate that differences in peak amplitudes beyond levels of full GR activation, as observed for 3-month old mice versus older ages, do not result in stronger effects on the cell cycle in NSPC. Moreover, we also show that the TDC exposure has little predictive power on the effect of CORT on the cell cycle in NSPC. These results indicate that the oscillatory CORT pattern itself is responsible for the effects on the cell cycle in NSPC. We propose that the hormone-free periods intrinsic of the ultradian GC oscillatory pattern are responsible for the effects on the cell cycle and possibly contribute to the preservation of GR + NSPC, which in contrast to their GR- counterparts, are able to sense these oscillatory patterns.

GC bind to the mineralocorticoid receptor (MR) with high affinity and to the GR with lower affinity [84, 85]. Previous work has indicated that adult hippocampal NSPC, do not express the MR [41, 44]. More recent single cell RNA-seq studies in hippocampal NSPC have confirmed GR (NR3C1) expression in hippocampal NSPC but failed to detect MR (NR3C2) expression [48]. Other studies have found that systemic treatment with the MR agonist aldosterone protects from ADX-induced cell death at low dose and partially inhibits ADX-induced cell proliferation at higher doses, indicating an intriguing and complex role for the MR in regulating progenitor cells in the GCL of the DG [86]. Importantly, due to its high affinity for CORT the MR is fully occupied at all diurnal levels, whereas the GR is fully activated only during the circadian peak phase, and thereby may be more relevant for GC oscillations [86, 87]. Between PND 1–14 in rodents a period of reduced adrenal and pituitary hormone release in response to specific stressors has been characterized (stress hyporesponsive period; SHRP) [88]. As such, it may be an interesting period to study a possible transition between developmental neurogenesis and AHN, which may take place between PND 7–14 in mice [89]. Regarding GR expression, the vast majority of NSPC present in organotypic cultures from PND 6 are GR^+^ (~80%) [90], in agreement with the relative abundance of GR + NSPC in the hippocampus of 3-month-old mice and in primary hippocampal NSPC cultures, as reported herein. Overall, these observations suggest a lack of developmental switch between the GR^+^ and GR^−^ NSPC populations in the neonatal period. However, as the focus of our current study was the regulation of AHN, we did not investigate this point further.

The use of high-CORT pellets may model pathological disruptions of GC oscillations, since these are frequently associated with increased nadir levels rather than decreased peak levels [6], and are consistent with increased neuronal survival, incorporation, and morphological rearrangements observed in DG neurons after chronic stress [91]. Others have compared the effect of high and low-CORT pellets [60, 61]. Specifically, these authors used pellets containing daily average CORT concentrations, which did not result in full GR activation [61]. Importantly, our results in vitro indicate that a maximal inhibition of NSPC proliferation is only achieved at GC concentrations that are compatible with full GR activation, suggesting that a complete induction of NSPC nonproliferative states may not be achieved using lower steady CORT levels. Supporting this hypothesis, constant incubation with submaximal [CORT] failed to induce the upregulation of Sgk-1 associated with GC-induced inhibition of NSPC proliferation [56]. Previous observations indicate that circadian peak levels in vivo in rats may be lower that the CORT concentrations we used to modeled them in vitro, and in the range of 5 × 10^−7^ M [6, 92]. This CORT concentration is expected to induce approximately 80% of the maximal effect on proliferation and cell cycle, based on dose–response curves presented in Fig. 4d. Therefore, we assume that the CORT conditions used in vitro reflect to a large extent those described in vivo before.

GC have direct effects on hippocampal NSPC in vivo, mediated by the GR. In particular, GR knockdown in neuroblasts accelerated their neuronal differentiation and migration [41]. Extending from these observations, genetic GR knockdown in 12-month-old GR^fl/wt^ mice using a split-Cre system that targets Type-1 NSPC specifically [50] resulted in increased Type-1 cell proliferation. Similarly, GR knockdown in 20-month-old wild-type mice using siRNAs also increased Type-1 cell proliferation. These two experimental approaches resulted in different levels of Type-1 cell activation. siRNA-mediated knockdown in 20-month-old wild-type mice was more efficacious than partial genetic disruption of the GR using the split-Cre system in 12-month-old GR^fl/wt^ mice. In these experiments, we did not directly address the fate of new cells generated from Type 1 cells. However, at the time of siRNAs injections, all Type-1 cells were GR+ and of the preferentially astrogenic Type-1β morphophenotype [55]. Although our data show a significant decrease in total Nestin-GFP+ cells with age, a general depletion of NSPC remains a controversial hypothesis. Previous work has indicated that Type-1 NSPC have a finite number of division cycles before they differentiate into astrocytes, thereby depleting the NSPC pool [27]. According to these findings, any factor that controls NSPC proliferation, i.e., GR expression, will also control NSPC depletion. In contrast, other authors have provided findings, that contradict this “disposable NSC” theory [34, 35, 93]. However, it has been proposed that these seemingly contradictory observations may be reconciled considering technical differences [36].

GC oscillations have been studied in vivo using automated intravenous blood sampling in rats [7], but this approach induces a stress reaction that disrupts circadian GC oscillations in mice [94]. Furthermore, it is difficult to eliminate indirect effects coming from other cell types present in the local environment [95]. In view of these limitations, we modeled GC oscillations in vitro using mouse hippocampal NSPC cultures, in which the direct effects of GC on NSPC can be readily characterized [41, 56]. Postnatal mouse hippocampal NSPC cultures are most commonly obtained from young animals, up to 8 weeks of age, due to optimal NSC numbers and proliferation capacities [96, 97]. Similarly, we used hippocampal NSPC obtained from young mice, which reflect the proportions of GR+ and GR− cells observed in 3-month-old mice in vivo. However, the relevance of this in vitro system for hippocampal NSPC present in older mice has to be interpreted with caution. Using this system, we compared the effects of oscillatory GC stimulation versus a continuous one [58], which may also reflect the situation observed in some hypercortisolaemic states in human [6]. We found that GC oscillations exert lasting effects on NSPC proliferation in vitro. GC oscillations maintained the sensitivity to inhibition of cell proliferation induced by GC in daughter cells, suggesting an epigenetic mechanism that may program NSPC proliferation. In contrast, daughter cells derived from NSPC exposed to continuous GC were desensitized to GC-induced inhibition of cell proliferation. These observations suggest that periods of prolonged exposure to continuous GC may result in lasting dis-inhibition of NSPC proliferation and in a decay of the NSPC pool, which may have negative consequences for long-term hippocampal plasticity [27]. Indeed, we could show an enhanced proliferation of Type-1 cells in vivo, 2 days after CORT pellet removal. Importantly, at this time point, endogenous GC oscillations remain inhibited, indicating that GC peaks originating by daily oscillations effectively suppress NSPC proliferation. This is compatible with the low NSPC proliferation rates observed in older mice, in which GR^+^ populations strongly predominate. Therefore, the preservation of a GR^+^ NSPC population is associated with the conservation of a nonproliferative NSPC pool in the aged DG.

In old mice, we observed an apparent reduction in 5-mC levels in Type-1 cells. In vitro, using NSPC cultures, we show that GC oscillations maintain DNMT expression levels within a controlled range in NSPC. These results are consistent with the concept that GC oscillations function to optimize steady-state gene expression, stabilizing responsive genes [9, 58]. In adolescent girls, alterations in GC oscillations are associated with changes in DNA methylation and reduced hippocampal volume [65]. We show here that GC oscillations induce strong hypermethylation effects in vitro on hippocampal NSPC, with 73% of the differentially methylated promoters being hypermethylated, which suggests that GC oscillations maintain specific DNA methylation states in NSPC. In total, 70 cell cycle-related gene promoters were differentially methylated by GC oscillations, indicating that their genome-wide promoter methylation effects may converge on the regulation of cell cycle in NSPC. The effects on methylation and gene expression were stronger in cells exposed to GC oscillations, likely reflecting an intrinsic effect of pulsatility. This latter conclusion is also supported by our results indicating that, at least when modeled in vitro, ultradian and circadian GC oscillations deliver different biological signals to the cell cycle in NSPC that may depend on periods of full GR activation [58]. Interestingly, some of the genome-wide changes on DNA methylation were lasting and persisted across NSPC generations. In agreement with our observations, exposure of the hippocampus to GC during embryonic development induced changes in DNA methylation in specific gene promoters, 24 h after the treatment. Similar to the results we describe here, the majority of these changes in promoter methylation were transient and only some promoters remained in their hypomethylated or hypermethylated state [98]. Within the lasting hypomethylated gene promoters, we identified a network of genes involved in Wnt signaling, a principal regulatory pathway in AHN [99]. Importantly, loss of the Wnt antagonist Dickkopf-1 (DKK1) in adult mice restores AHN, increases dendritic complexity of newborn granule neurons and counteracts age-associated cognitive decline [100]. Indeed, GC oscillations induced a stable expression profile, as compared to continuous GC, on four components of the Wnt signaling pathway (DKK3, GSK3β, CCND1, and β-catenin) when modeled in vitro on NSPC cultures. Our observations regarding the lasting effects on DNA methylation in NSPC in vitro suggest that GC oscillations may preserve certain components of the Wnt signaling pathway within a controlled expression range. Therefore, an exhaustive functional characterization of the regulation of Wnt signaling by GC oscillations and its possible consequences for other cellular processes such as cellular differentiation in NSPC warrants further investigation.

The use of accelerated senescence models, such as the SAMP8 mouse strain, provides an experimental alternative to the use of aged wild-type mice [101]. We found that AM and PM CORT levels were significantly elevated in untreated SAMP8 mice, supporting their use as model of circadian rhythm disturbances associated with pathological aging [74]. Disruption of circadian GC oscillations in SAMP8 mice induced by CORT pellet implantation was associated with lasting morphological changes in newborn neurons generated from NSPC at the time of pellet removal, as indicated by retroviral birth-dating. These morphological changes included increased dendritic complexity, spine numbers and relative numbers of immature spines, with seemingly opposite effects in control SAMR1 mice. The reduced complexity of newborn granule neurons we observed in SAMP8 is compatible with a delayed development of newborn neurons observed in the aging hippocampus [102] and with a GR-mediated regulation of newborn neuron development in the adult hippocampus [41]. The differences observed between SAM strains after disruption of GC oscillations may suggest the presence of alterations in endogenous GC levels in SAMP8 mice that affect the structural plasticity of newborn neurons in the adult hippocampus [95].

Recently, the concept of stress-induced stem cells has been introduced. This conceptualization proposes that the effects of stress on stem/progenitor cells in young individuals may predispose to disease later in life, affecting the renewal and regenerative potential of several tissues, thereby contributing to the development of metabolic and mental diseases [103]. In agreement with this idea, alterations in GC oscillations induced by severe physiological or psychological stress during aging may contribute to the effect of GC on NSPC and AHN [79, 104–107]. Moreover, recent data indicate that AHN confers resilience to chronic stress by inhibiting the activity of mature granule cells in the ventral DG^112^. Although the sustained presence of AHN in the aging human brain remains challenged by contrasting observations, most reports indicate a substantial decrease with age, albeit at different rates [17–20, 108]. Indeed, an age-associated exhaustion of the NSPC pool may explain some of the interindividual variations in cognitive and emotional states and resilience to stress-associated diseases related to aging [27, 109–112]. Importantly, recent observations have provided new and compelling evidence for the presence of AHN in the aged human hippocampus [33], suggesting that our observations could have implications for the understanding of human brain aging. In conclusion, our results indicate that GR expression and GC oscillations contribute to the preservation of distinct quiescent NSPC subpopulations during aging in vivo, providing a suitable mechanism for the aging-associated decline in AHN and highlight that a GC-controlled structural plasticity reserve remains available in the senescent brain.

## Methods

### Animal cohorts, CORT measurements, immunohistochemistry, and confocal microscopy

All animal procedures were approved by the Commission for Animal Welfare, at University of Amsterdam, Diputación Foral de Bizkaia and CSIC Madrid and were performed following EU regulations. Male 3, 6, 10, 14, and 18-month-old Nestin-GFP transgenic mice [113] (*n* = 5 per group) were used for experiments. These time points were selected based on start- and end-points of previously defined life-phases (mature adult, middle age, and old) in mice [114]. Mice were housed under standard laboratory cage conditions and kept under 12 h light/dark cycles (lights on at 08:00, lights off at 20:00) with ad libitum access to food and water. At weaning, all animals used were randomly allocated to the different experimental groups once their genotype/phenotype was established. At the indicated ages, tail blood was collected in a stress-free manner in ice-cold EDTA-coated tubes (Sarstedt, Etten-leur, The Netherlands) at 20:00 (PM) the night before and at 08:00 (PM) on the morning of perfusion, as described before [115]. Samples were kept on ice and subsequently centrifuged at 13,000 rpm for 15 min, blood plasma was stored at −20 °C. AM and PM plasma CORT levels were measured using a commercial radioimmunoassay kit (MP Biomedicals, Eindhoven, The Netherlands) as described before [115]. Animals were transcardially perfused at the indicated ages at 08:00 ± 0.3 h (Fig. 1u) with 4% paraformaldehyde in phosphate buffered saline (PBS) and brains were extracted, sectioned in 8 series of 40 μm-thick slices, ensuring a 280 nm separation between series used for individual inmunostainings as described before [41], using the following antibodies: polyclonal chicken anti-GFP (Abcam, 1:500), monoclonal mouse anti-GFAP (Chemicon, 1:1000) and polyclonal rabbit anti-GR (H300 Santa Cruz, 1:100) or polyclonal rabbit anti-Ki67 (Abcam, 1: 1000) in combination with goat anti-chicken Alexa488 (Invitrogen, 1:500), goat anti-mouse Alexa647 (Invitrogen, 1:500), and goat anti-rabbit Alexa568 (Invitrogen, 1:500), respectively. Proliferating cell nuclear antigen (PCNA) and 5-mC stainings required antigen retrieval, which was performed by heating brain sections in 0.1 M citrate buffer (pH 6.0) in a standard microwave (Samsung M6235) to a temperature of approximately 95 °C for 15 minutes (5 min at 800 W, 5 min at 400 W and 5 min at 200 W). Antibodies used were monoclonal mouse anti-PCNA (DAKO, 1:400) and monoclonal mouse anti-5-mC (Eurogentec, 1:500) in combination with goat anti-mouse Alexa647 (Invitrogen, 1:500) and if applicable combined with rabbit anti-GFAP (DAKO, 1:500) in combination goat anti-rabbit Alexa568. For the retroviral experiment stainings the following antibodies were used: polyclonal chicken anti-GFP (Abcam, 1:500), monoclonal mouse anti-NeuN (Chemicon, 1:1000) and polyclonal rabbit anti-GFAP (DAKO, 1:500) or polyclonal chicken anti-GFP (Abcam, 1:500), monoclonal mouse anti-NG2 (Millipore, 1:100) and polyclonal rabbit anti-Iba1 (Wako, 1:1000) in combination with goat anti-chicken Alexa488 (Invitrogen, 1:500), goat anti-mouse Alexa647 (Invitrogen, 1:500), and goat anti-rabbit Alexa568 (Invitrogen, 1:500), respectively. Sections were counterstained for DNA using Hoechst (Invitrogen. 1:20,000) to detect cell nuclei. Confocal microscopy was performed as described before using a Zeiss LSM510 laser scanning microscope [41]. Z-plane optical sectioning ranged from 150–500 nm. Hippocampal NSPC populations were quantified in the SGZ and GCL hereafter referred to as granular zone (GZ) and were either expressed in absolute numbers per mm^3^ GZ or in relative percentages of the total NSPC subpopulation. Staining intensity histograms were obtained from single confocal Z-planes using ImageJ, using the same imaging conditions for young and old animals.

### Generation of best-fit curves, population half-life calculations, correlations, and statistical analysis

Nonlinear (exponential decay) best-fit curves (*N*(*t*) *=* *N*_0_*e*^−*κt*^ *+* *N*_0_ with *N* as number of cells in cells/mm^3^ GZ, *t* as time in months and *κ* as the decay rate constant as a decimal) or linear (first order polynomial) decay curves (*N*(*t*) = *N*_0_ − *λt* with *N* as number of cells in cells/mm^3^ GZ, *t* as time in months and *λ* as the slope in cells/mm^3^ month^−1^), including their 95% confidence intervals were plotted on the numbers of (GR^+^ and GR^-^) Type-1 and Type-2 NSPC using Graphpad Prism 5.0 software. Non-linear (exponential) decay curves were tested for a significantly better fit than linear (first order polynomial) decay curves using an extra-sum-of-squares *F*-test and were considered significantly different if the *F*-test reached a *p* < 0.05. Subsequently, depending on the aforementioned extra-sum-of-squares *F*-test results, half-lives (or *t*_1/2_) were calculated for the either exponential (*t*_1/2_ = ln(1/2*)*/*κ*) or linear (*t*_1/2_ = *N*_3_/2/*λ*) curves from GR^+^ and GR^−^ Type-1 and Type-2 NSPC. For CORT concentrations versus NSPC population correlations a Pearson correlation analysis was used and were subsequently tested for significant deviation from a slope of 0 and were considered significantly different if *p* < 0.05. Graphpad Prism 5 software was used for the generation of best-fit curves and Pearson correlation analysis.

### Subcutaneous pellet implantation experiments

CORT levels were manipulated using slow release biodegradable carrier-binder pellets to various daily concentrations (vehicle, 12.5 mg/kg/day and 25 mg/kg/day, n = 4 per experimental group; Innovative Research of America), as described by others [60], albeit with some modifications. Pellets were implanted subcutaneously between the shoulder blades of Nestin-GFP animals under isoflurane anesthesia at 08:00 h on experimental day 1. When indicated, pellets were removed under isoflurane anesthesia at 08:00 h on experimental day8. PM and AM plasma CORT concentrations were determined on day 7/8 or 3 days after pellet removal (recovery group) on day 9/10 (Figure S4A). Immunohistochemical analysis was performed on 4 animals per experimental group, as described in the corresponding section.

### Stereotactic split-Cre lentiviral injections in 12-month-old GR floxed animals

Heterozygous transgenic mice with loxp sites flanking NR3C1 (GR) exon2 [51] (here named GR^fl/wt^), where purchased from The Jackson Laboratory (strain B6.129S6-Nr3c1tm2.1Ljm/J) and where compared to their wild-type littermates (here named GR^wt/wt^). Lentiviral-mediated GR knockout experiments were performed on 12-month-old male GR^fl/wt^ or GR^wt/wt^ mice. Animals were genotyped as described before [51], (Figure S4B). To induce recombination, a split-Cre lentiviral approach with the N-terminus of Cre under the expression of the GFAP promoter and the C-terminus of Cre under the Prominin1 promoter was used as previously described [50]. Linker structures on both termini enabled a functional Cre-recombinase and NSPC recombination was detected with a lentivirus expressing a floxed dsRED + STOP codon which upon recombination expresses eGFP [53] for which a specific anti-GFP staining was performed. 1.5 µl of a 1:1:1 ratio of these three lentiviruses were stereotactically delivered into the DG (anterior-posterior: −2.0, medial-lateral: ±1.5, dorsal-ventral: −2.0). 6 dpi 4 GR^fl/wt^ and GR^wt/wt^ animals were sacrificed. Native RFP and GFP signal was undetectable and we thus stained specifically for GFP to visualize cells with a radial glial-like Type-1 NSPC morphology expressing both Cre termini in combination with Ki67 to assess levels of proliferation, as described in the corresponding sections.

### Stereotactic siRNA injections in Nestin-GFP mice

For siRNA-mediated GR knockdown experiments, 20-month-old male Nestin-GFP mice underwent stereotaxic surgery, delivering 1 µl of a 40 µM mixture of 4 previously validated [41] siRNAs (FlexiTube GeneSolution, Qiagen, CAGACTCAGCATGGAGAATTA, AAGCGTGATGGACTTGTATAA, CAGTGGTGCGATAGCAACAAA, AAGGAAGGTCTGAAGAGCCAA) against the mouse GR (Nr3c1, Entrez gene ID: 14815) into the left DG or negative control siRNA (target sequence: AATTCTCCGAACGTGTCACGT; Qiagen) into the contralateral DG (anterior-posterior: −2.0, medial-lateral: ±1.5, dorsal-ventral: −2.0). Seventy-two hours after siRNA infusion, 6 animals were sacrificed by transcardial perfusion-fixation, brains were extracted and processed for immunohistochemistry as described in the corresponding section. Similarly, for naked siRNA uptake verification, male Nestin-GFP mice (*n* = 3) underwent the same procedure in which negative control Cy3-labeled siRNA (siNC^Cy3^; Allstars negative control siRNA; Cat. No. SI03650318, Qiagen) was delivered. These animals were sacrificed 24 h after (1 dpi) siRNA infusion as described above. For naked siRNA knockdown validation, male Nestin-GFP mice (*n* = 3) were injected with siNC (Allstars negative control siRNA; Cat. No. SI03650318, Qiagen) and siRNA directed against GFP (positive silencing control GFP-22 siRNA, Cat. No. 0001022064, Qiagen). Naked siRNA knockdown validation animals were sacrificed 3 dpi. After brain slices were obtained, they were stained for DNA using Hoechst and native GFP and Cy3 colocalization or native GFP intensity levels were measured using the Zeiss LSM510 confocal as described in the corresponding section.

### Retrovirus production

RV-GFP was done as described before [116]. HEK293T cells were co-transfected with pCAG-GFP, pCMV-GP, and pCMV-VSV-G (3:2:1) plasmids by calcium-phosphate precipitation. The media containing retrovirus was collected 48 h after transfection. Cell debris was removed from the supernatant by centrifugation at 3200×*g* for 10 min and filtration through a 0.22 μm filter. The retrovirus was concentrated by ultra-centrifugation at 160,000×*g* for 2 h (Sorvall WX Ultracentrifuge and SureSpin 630 swinging bucket rotor; Thermo Fisher Scientific, Waltham, MA, USA). The retroviral pellet was resuspended in 200 μl phosphate buffered saline (PBS; Sigma-Aldrich, St. Louis, MO, USA), aliquoted and stored at −80 °C. The titer was at 10^5^ colony forming units.

### CORT pellet implantation and retrovirus-GFP labeling of newborn cells in SAMP8 and SAMR1 mice

4-month-old male senescence-accelerated mouse-prone 8 (SAMP8) and control senescence-accelerated mouse-resistant 1 (SAMR1) mice received subcutaneous 12.5 mg/kg/day CORT and control pellets as described above for 7 days. Subsequently pellets were removed and the animals were allowed to recover for 2 days before they underwent stereotaxic injection of 1.5 μl of a retrovirus suspension prepared as described in the previous section. 28 dpi of the retrovirus mice (*n* = 4 per group) were sacrificed by transcardial perfusion-fixation, brains were extracted and processed for immunohistochemistry as described in the corresponding section. Another cohort of SAMR1 and SAMP8 animals was sacrificed either at day 7 after pellet implantation, or at day 9, 2 days after pellet removal without receiving retroviral injections to assess hippocampal Ki67 expression. The GFP signal from RV-GFP^+^/NeuN^+^/Iba1^−^/NG2^−^ cells was traced using ImageJ and Sholl analyses were performed as described before [41]. Furthermore, from RV-GFP^+^/NeuN^+^/Iba1^−^/NG2- newborn cells the spine density and morphology analyses were performed in using the software package Neuron Studio on secondary/tertiary dendritic segments, as described before [41].

### Cell culture, CORT treatments, and CORT measurements

Primary hippocampal NSPC cultures were prepared and maintained in culture flasks in DMEM/F-12 medium supplemented with 5% charcoal-stripped fetal bovine serum (FBS, Atlanta Biologicals), N2 supplement, (Invitrogen), bovine pituitary extract (BPE, Invitrogen), recombinant-human-EGF (20 ng/mL, Sigma) and recombinant-human-FGF (10 ng/mL, Sigma), as described before [41]. NSPC were seeded the day before the start of the treatments. CORT (corticosterone, Sigma-Alrdich) was dissolved in (vehicle) and added freshly to NSPC medium to a final concentration of 1 × 10^−6^M (except stated otherwise) prior to incubation. CORT oscillations were modeled in vitro as previously described [58]. Briefly, pulsatile treatment consisted of 30-min long incubation with either vehicle or CORT, interspaced with 30 min-long incubations with hormone-free medium, mimicking CORT ultradian pulses. NSPC were exposed to this pulsatile treatment for 12 h, followed by a 12-h long incubation with hormone-free medium, to model circadian oscillations. The continuous CORT condition consisted of 30 min-long cycles of incubation with CORT for 24 h, without interspaced hormone-free periods (Fig. S4N, O). Additional groups consisted of pulsatile treatment with 2 μM CORT, continuous 0.25 μM CORT, and 1 μM CORT for 12 h followed by 12 h hormone-free periods to mimic circadian rhythmicity (see also Fig. S5A, B). Starting after a 72 h initial treatment, the washout period (recovery) consisted of a 24 h-long incubation with hormone-free medium. When indicated, NSPC were treated during the last 6 h of the washout period with 10^−6^ M CORT or vehicle, to model the effects on further exposure to CORT. Treatment schemes are depicted in Fig. S4N. Efficient washout and stability of CORT during the experiment (Fig. S4O) was analyzed by collecting samples every 30 min during both oscillating and continuous CORT treatment and CORT concentrations were determined using a commercial radioimmunoassay kit (MP Biomedicals, Eindhoven, The Netherlands) as described above.

### Immunocytochemistry

Immunocytochemistry was carried out as described before [41]. Briefly, cells were rinsed three times with PBS and fixed in 4% PFA in PBS for 30 min. The fixative was then removed and cells were rinsed three times for 5 min with PBS. For detection of proliferation, cells were blocked in blocking buffer (1× TBS/1% skimmed milk powder) for 60 min and incubated for 1 h at room temperature and then overnight at 4 °C with polyclonal rabbit anti-Ki67 (Abcam, 1:1000) diluted in 0.25% gelatin/0.5% Triton X-100 (Supermix). The day after, cells were rinsed three times for 5 min in PBS, incubated with donkey anti-rabbit Alexa488 (Invitrogen, 1:1000) for 1 h at room temperature, rinsed three times for 5 min in PBS and mounted in Vectashield Mounting Medium with DAPI (Vector Laboratories).

To assess GR immunoreactivity in Ki67-expressing NSPC, blocking buffer was applied for 60 min before cells were incubated for 1 h at room temperature and then overnight at 4 °C with a polyclonal mouse anti-Ki67 (Novocastra, 1:200) and polyclonal rabbit anti-GR (H300 Santa Cruz, 1:200) antibody diluted in Supermix. The day after, cells were rinsed three times for 5 min in PBS, incubated with goat anti-mouse Alexa568 (Invitrogen, 1:1000) and donkey anti-rabbit Alexa488 (Invitrogen, 1:1000) for 1 h at room temperature, rinsed three times for 5 min in PBS and mounted in Vectashield Mounting Medium with DAPI (Vector Laboratories). Images were acquired using a Leica CTR5500 microscope with the Leica MM AF program (MetaMorph, version 1.6.0).

### Quantitative real time PCR

RNA was isolated using TRIzol reagent (Life Technologies) according to the manufacturers’ protocol. For mRNA qPCRs, cDNA was synthetized using a superscript II reverse transcriptase (Life Technologies) according to the manufacturers’ protocol. Quantitative real time PCR were performed, as described before [41], using SYBR green (Applied Biosystems) and the following primer sequences: α-tubulin (for normalization) forward: CCCTCGCCTTCTAACGCGTTGC, reverse: TGGTCTTGTCACTTGGCATCTGGC; DNMT1 forward: AGGCGCGTCATGGGTGCTAC, reverse: GGCGGCGCTTCATGGCATTC; DNMT3a forward: GCCAAGAAACCCAGAAAGAGC, reverse: GTGACATTGAGGCTCCCACA; DNMT3b forward: GCGTCAGTACCCCATCAGTT, reverse: ATCTTTCCCCACACGAGGTC; DKK3 forward: CACAATGAGACCAGCACGGA, reverse: GGCTCCTCTTGCCTTCTTCAT; GSK3β forward: CCCTCAAATCAAGGCACATCC, reverse: TTGGGTCCCGCAATTCATCG; CCND1 forward: GCCATGACTCCCCACGATTT, reverse: CTACCATGGAGGGTGGGTTG; and β-catenin forward: GAACAGGGTGCTATTCCACGA, reverse: TGGAGAGCTCCAGTACACCC; Hes5 forward: AGCAAAGCCTTCGCCGC, reverse: CCGCTGGAAGTGGTAAAGCA; SGK1 forward: TGGTGTCTTGGGGCTGTCCTGT, reverse: GCCTTCCAGGAGTGTCCTTGC.

### Flow cytometry analysis of cell cycle using propidium iodide

NSPC were trypsinized (Trypzean, Lonza) for 5 min and fixed by slowly adding cold 70% ethanol (−20 °C) and were then left overnight at 4 °C. Subsequently, cells were washed twice with PBS for 5 min and treated for 20 min with RNAse (100 μg/ml; Sigma-Aldrich) and incubated for 20 min at room temperature with a mix containing propidium iodide (5 µg/ml; Sigma-Alrdich), 0.1% sodium citrate and Triton-X100 (0.1%) in PBS. Cells were sorted using a FACSAria^™^ III system (BD) with 488 nm excitation laser. Propidium iodide was detected within the PE/Texas Red channel with a 610/10 bandpass filter. At least 9000 cells were analyzed per sample and only single cells were included in the analysis. The fluorescence intensity of each single cell indicating total DNA content was used to classify cells in the G0/G1 (2N), S (>2N), M (4N) phase or apoptotic cells (<2N). FACS histograms were plot-fitted using the G2/G1 fixed method using Multicycle AV and FCS express (De Novo Software).

### Global cytosine methylation analysis

Global DNA methylation was measured using MBD-isolated Genome Sequencing, essentially as described [68]. Briefly, NSPC were trypsinized (Trypzean, Lonza) for 5 min, spun down for 3 min at 300×*g* and total DNA was extracted using a GenElute^™^ Mammalian Genomic DNA Miniprep Kit (Sigma-Aldrich) following the manufacturer’s protocol. Global levels of DNA methylation were measured using a Methylamp^™^ Global DNA Methylation Quantification Ultra Kit (Epigentek) according to the manufacturer’s protocol. Data were normalized to global DNA methylation levels of vehicle treated NSPC, as indicated.

### Methylated DNA sample preparation and quality control

DNA was isolated from NSPC as described above. DNA concentration was determined on a Fluostar Optima plate reader (BMG Labtech) with the Quant-iTTM Picogreen^®^ dsDNA assay kit (Invitrogen) at 480/520 nm. Concentration was determined using smear analysis on an Agilent 2100 Bioanalyzer (Agilent Technologies) and checked for degradation. Samples (*n* = 3) for each experimental condition were pooled into a single sample for further processing.

### Methylated DNA fragmentation and MBD2-capture

DNA fragmentation was performed on a Covaris S2 Focused ultrasonicator with the following settings: duty cycle 10%, intensity 5, 200 cycles per burst during 190 s to obtain fragments with an average length of 200 bp. The power mode was set to frequency sweeping, temperature 6–8 °C and water level 12. A maximum of 3 μg DNA was dissolved in 130 μl TE and loaded in a microtube with AFA intensifier (Covaris). DNA was then analyzed on the Agilent 2100 Bioanalyzer (Agilent Technologies) and fragment distribution was analyzed on a high sensitivity DNA chip. Methylated DNA was captured using the MethylCap kit (Diagenode). The concentrations of the fragmented and captured DNA was determined on a Fluostar Optima plate reader (BMG Labtech) with the Quant-iTTM Picogreen^®^ dsDNA assay kit (Invitrogen) at 480/520 nm. A second quality control was performed after fragmentation on an Agilent 2100 HS DNA chip.

### Methylated DNA library preparation, amplification and sequencing

A methylated DNA library was prepared, amplified and sequenced using a modified version of the “multiplexed paired end ChIP protocol” (Illumina) [68], using the DNA Sample Prep Master Mix Set 1 (NEB) in combination with the Multiplexing Sample Preparation Oligo Kit (Illumina). The library was prepared from 250 ng of fragmented DNA on an Apollo 324 NGS Library Prep System (IntegenX) with a PrepXDNA Library Kit (Wafergen Biosystems) according to the kit’s protocol. Library amplification was done according to the multiplexed paired end ChIP protocol including the indexes from Multiplexing Sample Preparation Oligo Kit (Illumina). Smaller fragments were removed when necessary using a 2% agarose gel (Low Range Ultra agarose; Biorad) in combination with a 1 kb Plus ladder (Invitrogen). 300 bp + /−50 bp fragments were excised and eluted on a Qiagen Gel Extraction Kit column (Qiagen), then eluted in 23 μl EB and 1 μl from there was run on an Agilent 2100 HS DNA chip. DNA concentration was determined using smear analysis on an Agilent 2100 Bioanalyzer and samples were diluted to 10 nM. DNA fragments were sequenced using the Hi-Seq 2000 Massive Parallel Sequencer system (Illumina) with 2 × 51 + 7(index) sequencing cycles. Initial quality assessment was based on data passing the Illumina Chastity filter control. Subsequently, the reads containing adapters and/or Phix control signal were removed. A second quality assessment was based on the remaining reads using the FASTQC quality control tool version 0.10.0.

### DNA methylation base scaling and mapping

FASTQ sequence reads were generated using the Illumina Casava pipeline version 1.8.0. The paired end 51 bp sequence reads were mapped using Bowtie software v0.12.7, as described [117]. The Bowtie parameters were set to 0 mismatches in the seed (first 28 nucleotides). Only unique paired reads were retained and both fragments must be located within 400 bp of each other on the mouse reference genome build NCBI37/mm9. Regions within −2000 and +500 bp from a TSS were considered as gene promoters.

### Bio-informatics and statistics

Dose–response curves were created using Graphpad Prism 5.0 and statistically compared with an *F*-test. Heatmaps were generated using the UHC option in MultiExperiment Viewer v4.9 (TM4). GO analysis was performed using the Genecodis GO algorithm hypergeometrically testing for significantly overrepresented processes (FDR corrected *p* < 0.05) as described [118], and functional network predictions were produced using the GeneMANIA algorithm [119]. The H2G2 genome browser (NXT-Dx) was used to explore the mapped MBD2 read density. All other comparisons were statistically tested using an unpaired two-tailed Student’s *t* test, one-way analysis of variance (ANOVA) test with Tukey’s post-test when more than two groups were compared, or two-way ANOVA test with a Bonferroni post-test when more than two groups with two independent variables were compared. The sample sizes were chosen based on previously observed effect sizes and calculated with a sigma of 0.2, alpha of 0.05 to obtain a power of at least 0.8 using the G Power software [120]. No samples or animals were excluded from our analyses. Statistical analyses were performed using GraphPad Prism 5.0.

## Supplementary information

Supplemental text

Supplemental Figure 1

Supplemental Figure 2

Supplemental Figure 3

Supplemental Figure 4

Supplemental Figure 5

Supplemental Figure 6

Supplemental Figure 7

Supplemental Figure 8

Supplemental Figure 9

Supplemental Table 1

Supplemental Table 2

## References

[1] Tucker-Drob EM (2011). Neurocognitive functions and everyday functions change together in old age. Neuropsychology.

[2] Cole JH, Ritchie SJ, Bastin ME, Valdes Hernandez MC, Munoz Maniega S, Royle N (2018). Brain age predicts mortality. Mol Psychiatry.

[3] Oster H, Challet E, Ott V, Arvat E, de Kloet ER, Dijk DJ (2017). The functional and clinical significance of the 24-hour rhythm of circulating glucocorticoids. Endocr Rev.

[4] Abercrombie HC, Giese-Davis J, Sephton S, Epel ES, Turner-Cobb JM, Spiegel D (2004). Flattened cortisol rhythms in metastatic breast cancer patients. Psychoneuroendocrinology.

[5] McEwen BS (2003). Interacting mediators of allostasis and allostatic load: towards an understanding of resilience in aging. Metabolism.

[6] Lightman SL, Conway-Campbell BL (2010). The crucial role of pulsatile activity of the HPA axis for continuous dynamic equilibration. Nat Rev Neurosci.

[7] Walker JJ, Spiga F, Waite E, Zhao Z, Kershaw Y, Terry JR (2012). The origin of glucocorticoid hormone oscillations. PLoS Biol.

[8] Schmidt MV, Enthoven L, van der Mark M, Levine S, de Kloet ER, Oitzl MS (2003). The postnatal development of the hypothalamic-pituitary-adrenal axis in the mouse. Int J Dev Neurosci.

[9] Stavreva DA, Coulon A, Baek S, Sung MH, John S, Stixova L (2015). Dynamics of chromatin accessibility and long-range interactions in response to glucocorticoid pulsing. Genome Res.

[10] Conway-Campbell BL, Sarabdjitsingh RA, McKenna MA, Pooley JR, Kershaw YM, Meijer OC (2010). Glucocorticoid ultradian rhythmicity directs cyclical gene pulsing of the clock gene period 1 in rat hippocampus. J Neuroendocrinol.

[11] Dalm S, Enthoven L, Meijer OC, van der Mark MH, Karssen AM, de Kloet ER (2005). Age-related changes in hypothalamic-pituitary-adrenal axis activity of male C57BL/6J mice. Neuroendocrinology.

[12] Liston C, Cichon JM, Jeanneteau F, Jia Z, Chao MV, Gan WB (2013). Circadian glucocorticoid oscillations promote learning-dependent synapse formation and maintenance. Nat Neurosci.

[13] Ikeda Y, Kumagai H, Skach A, Sato M, Yanagisawa M (2013). Modulation of circadian glucocorticoid oscillation via adrenal opioid-CXCR7 signaling alters emotional behavior. Cell.

[14] Gilhooley MJ, Pinnock SB, Herbert J (2011). Rhythmic expression of per1 in the dentate gyrus is suppressed by corticosterone: implications for neurogenesis. Neurosci Lett.

[15] Altman J, Das GD (1965). Autoradiographic and histological evidence of postnatal hippocampal neurogenesis in rats. J Comp Neurol.

[16] Kempermann G, Jessberger S, Steiner B, Kronenberg G (2004). Milestones of neuronal development in the adult hippocampus. Trends Neurosci.

[17] Spalding KL, Bergmann O, Alkass K, Bernard S, Salehpour M, Huttner HB (2013). Dynamics of hippocampal neurogenesis in adult humans. Cell.

[18] Kempermann G, Gage FH, Aigner L, Song H, Curtis MA, Thuret S et al. Human adult neurogenesis: evidence and remaining questions. Cell Stem Cell. 2018;23:25–30. 10.1016/j.stem.2018.04.004 PMC6035081 29681514

[19] Boldrini M, Fulmore CA, Tartt AN, Simeon LR, Pavlova I, Poposka V (2018). Human Hippocampal Neurogenesis Persists throughout Aging. Cell Stem Cell.

[20] Eriksson PS, Perfilieva E, Bjork-Eriksson T, Alborn AM, Nordborg C, Peterson DA (1998). Neurogenesis in the adult human hippocampus. Nat Med.

[21] Knoth R, Singec I, Ditter M, Pantazis G, Capetian P, Meyer RP (2010). Murine features of neurogenesis in the human hippocampus across the lifespan from 0 to 100 years. PLoS One.

[22] Kuhn HG, Dickinson-Anson H, Gage FH (1996). Neurogenesis in the dentate gyrus of the adult rat: age-related decrease of neuronal progenitor proliferation. J Neurosci.

[23] Cameron HA, McKay RD (1999). Restoring production of hippocampal neurons in old age. Nat Neurosci.

[24] Lazic SE (2012). Modeling hippocampal neurogenesis across the lifespan in seven species. Neurobiol Aging.

[25] Leuner B, Kozorovitskiy Y, Gross CG, Gould E (2007). Diminished adult neurogenesis in the marmoset brain precedes old age. Proc Natl Acad Sci USA.

[26] Ben Abdallah NM, Slomianka L, Lipp HP (2007). Reversible effect of X-irradiation on proliferation, neurogenesis, and cell death in the dentate gyrus of adult mice. Hippocampus.

[27] Encinas JM, Michurina TV, Peunova N, Park JH, Tordo J, Peterson DA (2011). Division-coupled astrocytic differentiation and age-related depletion of neural stem cells in the adult hippocampus. Cell Stem Cell.

[28] Mathews KJ, Allen KM, Boerrigter D, Ball H, Shannon Weickert C, Double KL (2017). Evidence for reduced neurogenesis in the aging human hippocampus despite stable stem cell markers. Aging Cell.

[29] Dennis CV, Suh LS, Rodriguez ML, Kril JJ, Sutherland GT (2016). Human adult neurogenesis across the ages: an immunohistochemical study. Neuropathol Appl Neurobiol.

[30] Kippin TE, Martens DJ, van der Kooy D (2005). p21 loss compromises the relative quiescence of forebrain stem cell proliferation leading to exhaustion of their proliferation capacity. Genes Dev.

[31] Furutachi S, Matsumoto A, Nakayama KI, Gotoh Y (2013). p57 controls adult neural stem cell quiescence and modulates the pace of lifelong neurogenesis. EMBO J.

[32] Toda T, Parylak SL, Linker SB, Gage FH The role of adult hippocampal neurogenesis in brain health and disease. Mol Psychiatry. 2018;24:67–87. 10.1038/s41380-018-0036-2 PMC6195869 29679070

[33] Moreno-Jimenez EP, Flor-Garcia M, Terreros-Roncal J, Rabano A, Cafini F, Pallas-Bazarra N et al. Adult hippocampal neurogenesis is abundant in neurologically healthy subjects and drops sharply in patients with Alzheimer's disease. Nat Med. 2019;25:554–560. 10.1038/s41591-019-0375-9 30911133

[34] Kempermann G (2011). The pessimist's and optimist's views of adult neurogenesis. Cell.

[35] Bonaguidi MA, Wheeler MA, Shapiro JS, Stadel RP, Sun GJ, Ming GL (2011). In vivo clonal analysis reveals self-renewing and multipotent adult neural stem cell characteristics. Cell.

[36] Lugert S, Taylor V (2011). Neural stem cells: disposable, end-state glia?. Cell Stem Cell.

[37] Lucassen PJ, Toni N, Kempermann G, Frisen J, Gage FH, Swaab DF. Limits to human neurogenesis-really? Mol Psychiatry 2019. 10.1038/s41380-018-0337-5 [Epub ahead of print]. 10.1038/s41380-018-0337-5 PMC7515796 30617274

[38] Cameron HA, Gould E (1994). Adult neurogenesis is regulated by adrenal steroids in the dentate gyrus. Neuroscience.

[39] Montaron MF, Drapeau E, Dupret D, Kitchener P, Aurousseau C, Le Moal M (2006). Lifelong corticosterone level determines age-related decline in neurogenesis and memory. Neurobiol Aging.

[40] Yu S, Patchev AV, Wu Y, Lu J, Holsboer F, Zhang JZ (2010). Depletion of the neural precursor cell pool by glucocorticoids. Ann Neurol.

[41] Fitzsimons CP, van Hooijdonk LW, Schouten M, Zalachoras I, Brinks V, Zheng T (2013). Knockdown of the glucocorticoid receptor alters functional integration of newborn neurons in the adult hippocampus and impairs fear-motivated behavior. Mol Psychiatry.

[42] Montaron MF, Petry KG, Rodriguez JJ, Marinelli M, Aurousseau C, Rougon G (1999). Adrenalectomy increases neurogenesis but not PSA-NCAM expression in aged dentate gyrus. Eur J Neurosci.

[43] Brunson KL, Baram TZ, Bender RA (2005). Hippocampal neurogenesis is not enhanced by lifelong reduction of glucocorticoid levels. Hippocampus.

[44] Garcia A, Steiner B, Kronenberg G, Bick-Sander A, Kempermann G (2004). Age-dependent expression of glucocorticoid- and mineralocorticoid receptors on neural precursor cell populations in the adult murine hippocampus. Aging Cell.

[45] Jhaveri DJ, O'Keeffe I, Robinson GJ, Zhao QY, Zhang ZH, Nink V (2015). Purification of neural precursor cells reveals the presence of distinct, stimulus-specific subpopulations of quiescent precursors in the adult mouse hippocampus. J Neurosci.

[46] Steiner B, Klempin F, Wang L, Kott M, Kettenmann H, Kempermann G (2006). Type-2 cells as link between glial and neuronal lineage in adult hippocampal neurogenesis. Glia.

[47] Encinas JM, Vaahtokari A, Enikolopov G (2006). Fluoxetine targets early progenitor cells in the adult brain. Proc Natl Acad Sci USA.

[48] Shin J, Berg DA, Zhu Y, Shin JY, Song J, Bonaguidi MA (2015). Single-cell RNA-Seq with waterfall reveals molecular cascades underlying adult neurogenesis. Cell Stem Cell.

[49] Murray F, Smith DW, Hutson PH (2008). Chronic low dose corticosterone exposure decreased hippocampal cell proliferation, volume and induced anxiety and depression like behaviours in mice. Eur J Pharmacol.

[50] Beckervordersandforth R, Deshpande A, Schaffner I, Huttner HB, Lepier A, Lie DC (2014). In vivo targeting of adult neural stem cells in the dentate gyrus by a split-cre approach. Stem Cell Rep.

[51] Brewer JA, Khor B, Vogt SK, Muglia LM, Fujiwara H, Haegele KE (2003). T-cell glucocorticoid receptor is required to suppress COX-2-mediated lethal immune activation. Nat Med.

[52] DiFiglia M, Sena-Esteves M, Chase K, Sapp E, Pfister E, Sass M (2007). Therapeutic silencing of mutant huntingtin with siRNA attenuates striatal and cortical neuropathology and behavioral deficits. Proc Natl Acad Sci USA.

[53] Zomer A, Maynard C, Verweij FJ, Kamermans A, Schafer R, Beerling E (2015). In vivo imaging reveals extracellular vesicle-mediated phenocopying of metastatic behavior. Cell.

[54] Mittelstadt PR, Monteiro JP, Ashwell JD (2012). Thymocyte responsiveness to endogenous glucocorticoids is required for immunological fitness. J Clin Invest.

[55] Gebara E, Bonaguidi MA, Beckervordersandforth R, Sultan S, Udry F, Gijs PJ (2016). Heterogeneity of radial glia-like cells in the adult hippocampus. Stem Cells.

[56] Anacker C, Cattaneo A, Musaelyan K, Zunszain PA, Horowitz M, Molteni R (2013). Role for the kinase SGK1 in stress, depression, and glucocorticoid effects on hippocampal neurogenesis. Proc Natl Acad Sci USA.

[57] Mulatero P, Panarelli M, Schiavone D, Rossi A, Mengozzi G, Kenyon CJ (1997). Impaired cortisol binding to glucocorticoid receptors in hypertensive patients. Hypertension.

[58] Stavreva DA, Wiench M, John S, Conway-Campbell BL, McKenna MA, Pooley JR (2009). Ultradian hormone stimulation induces glucocorticoid receptor-mediated pulses of gene transcription. Nat Cell Biol.

[59] Lugert S, Basak O, Knuckles P, Haussler U, Fabel K, Gotz M (2010). Quiescent and active hippocampal neural stem cells with distinct morphologies respond selectively to physiological and pathological stimuli and aging. Cell Stem Cell.

[60] Sarabdjitsingh RA, Isenia S, Polman A, Mijalkovic J, Lachize S, Datson N (2010). Disrupted corticosterone pulsatile patterns attenuate responsiveness to glucocorticoid signaling in rat brain. Endocrinology.

[61] Sarabdjitsingh RA, Spiga F, Oitzl MS, Kershaw Y, Meijer OC, Lightman SL (2010). Recovery from disrupted ultradian glucocorticoid rhythmicity reveals a dissociation between hormonal and behavioural stress responsiveness. J Neuroendocrinol.

[62] Ponti G, Obernier K, Guinto C, Jose L, Bonfanti L, Alvarez-Buylla A (2013). Cell cycle and lineage progression of neural progenitors in the ventricular-subventricular zones of adult mice. Proc Natl Acad Sci USA.

[63] Ma DK, Jang MH, Guo JU, Kitabatake Y, Chang ML, Pow-Anpongkul N (2009). Neuronal activity-induced Gadd45b promotes epigenetic DNA demethylation and adult neurogenesis. Science.

[64] Wu H, Coskun V, Tao J, Xie W, Ge W, Yoshikawa K (2010). Dnmt3a-dependent nonpromoter DNA methylation facilitates transcription of neurogenic genes. Science.

[65] Davis EG, Humphreys KL, McEwen LM, Sacchet MD, Camacho MC, MacIsaac JL (2017). Accelerated DNA methylation age in adolescent girls: associations with elevated diurnal cortisol and reduced hippocampal volume. Transl Psychiatry.

[66] Goll MG, Bestor TH (2005). Eukaryotic cytosine methyltransferases. Annu Rev Biochem.

[67] Bose R, Moors M, Tofighi R, Cascante A, Hermanson O, Ceccatelli S (2010). Glucocorticoids induce long-lasting effects in neural stem cells resulting in senescence-related alterations. Cell Death Dis.

[68] Serre D, Lee BH, Ting AH (2010). MBD-isolated Genome Sequencing provides a high-throughput and comprehensive survey of DNA methylation in the human genome. Nucleic Acids Res.

[69] Ohta A, Akiguchi I, Seriu N, Ohnishi K, Yagi H, Higuchi K (2002). Deterioration in learning and memory of inferential tasks for evaluation of transitivity and symmetry in aged SAMP8 mice. Hippocampus.

[70] Soriano-Canton R, Perez-Villalba A, Morante-Redolat JM, Marques-Torrejon MA, Pallas M, Perez-Sanchez F (2015). Regulation of the p19(Arf)/p53 pathway by histone acetylation underlies neural stem cell behavior in senescence-prone SAMP8 mice. Aging Cell.

[71] Diaz-Moreno M, Hortiguela R, Goncalves A, Garcia-Carpio I, Manich G, Garcia-Bermudez E (2013). Abeta increases neural stem cell activity in senescence-accelerated SAMP8 mice. Neurobiol Aging.

[72] Gang B, Yue C, Han N, Xue H, Li B, Sun L (2011). Limited hippocampal neurogenesis in SAMP8 mouse model of Alzheimer's disease. Brain Res.

[73] Yanai S, Endo S (2016). Early onset of behavioral alterations in senescence-accelerated mouse prone 8 (SAMP8). Behav Brain Res.

[74] Pang KC, Miller JP, Fortress A, McAuley JD (2006). Age-related disruptions of circadian rhythm and memory in the senescence-accelerated mouse (SAMP8). Age.

[75] Yagi H, Katoh S, Akiguchi I, Takeda T (1988). Age-related deterioration of ability of acquisition in memory and learning in senescence accelerated mouse: SAM-P/8 as an animal model of disturbances in recent memory. Brain Res.

[76] van Praag H, Schinder AF, Christie BR, Toni N, Palmer TD, Gage FH (2002). Functional neurogenesis in the adult hippocampus. Nature.

[77] Klempin F, Kronenberg G, Cheung G, Kettenmann H, Kempermann G (2011). Properties of doublecortin-(DCX)-expressing cells in the piriform cortex compared to the neurogenic dentate gyrus of adult mice. PLoS One.

[78] Brandt MD, Maass A, Kempermann G, Storch A (2010). Physical exercise increases Notch activity, proliferation and cell cycle exit of type-3 progenitor cells in adult hippocampal neurogenesis. Eur J Neurosci.

[79] Fitzsimons CP, Herbert J, Schouten M, Meijer OC, Lucassen PJ, Lightman S (2016). Circadian and ultradian glucocorticoid rhythmicity: Implications for the effects of glucocorticoids on neural stem cells and adult hippocampal neurogenesis. Front Neuroendocrinol.

[80] Schoenfeld TJ, Gould EStress (2012). stress hormones, and adult neurogenesis. Exp Neurol.

[81] Meaney MJ, Aitken DH, Sharma S, Viau V (1992). Basal ACTH, corticosterone and corticosterone-binding globulin levels over the diurnal cycle, and age-related changes in hippocampal type I and type II corticosteroid receptor binding capacity in young and aged, handled and nonhandled rats. Neuroendocrinology.

[82] Oomen CA, Mayer JL, de Kloet ER, Joels M, Lucassen PJ (2007). Brief treatment with the glucocorticoid receptor antagonist mifepristone normalizes the reduction in neurogenesis after chronic stress. Eur J Neurosci.

[83] Kim JB, Ju JY, Kim JH, Kim TY, Yang BH, Lee YS (2004). Dexamethasone inhibits proliferation of adult hippocampal neurogenesis in vivo and in vitro. Brain Res.

[84] Rousseau G, Baxter JD, Funder JW, Edelman IS, Tomkins GM (1972). Glucocorticoid and mineralocorticoid receptors for aldosterone. J Steroid Biochem.

[85] Reul JM, de Kloet ER (1985). Two receptor systems for corticosterone in rat brain: microdistribution and differential occupation. Endocrinology.

[86] Montaron MF, Piazza PV, Aurousseau C, Urani A, Le Moal M, Abrous DN (2003). Implication of corticosteroid receptors in the regulation of hippocampal structural plasticity. Eur J Neurosci.

[87] Reul JM, van den Bosch FR, de Kloet ER (1987). Differential response of type I and type II corticosteroid receptors to changes in plasma steroid level and circadian rhythmicity. Neuroendocrinology.

[88] Vázquez DML, Levine S. Hypothalamic-pituitary-adrenal axis in postnatal life. In: Steckler TK,NH, Reul, JMHM, editors. Handbook of Stress and the Brain. Part 2: Stress: Integrative and Clinical Aspects, vol. 15. Elsevier, Amsterdam, The Netherlands; 2005, p. 3–21.

[89] Nicola Z, Fabel K, Kempermann G (2015). Development of the adult neurogenic niche in the hippocampus of mice. Front Neuroanat.

[90] Fitzsimons CP, Ahmed S, Wittevrongel CF, Schouten TG, Dijkmans TF, Scheenen WJ (2008). The microtubule-associated protein doublecortin-like regulates the transport of the glucocorticoid receptor in neuronal progenitor cells. Mol Endocrinol.

[91] De Miguel Z, Haditsch U, Palmer TD, Azpiroz A, Sapolsky RM. Adult-generated neurons born during chronic social stress are uniquely adapted to respond to subsequent chronic social stress. Mol Psychiatry. 2018. 10.1038/s41380-017-0013-1 [Epub ahead of print]. 10.1038/s41380-017-0013-1 29311652

[92] Qian X, Droste SK, Gutierrez-Mecinas M, Collins A, Kersante F, Reul JM (2011). A rapid release of corticosteroid-binding globulin from the liver restrains the glucocorticoid hormone response to acute stress. Endocrinology.

[93] Hattiangady B, Shetty AK (2008). Aging does not alter the number or phenotype of putative stem/progenitor cells in the neurogenic region of the hippocampus. Neurobiol Aging.

[94] Teilmann AC, Jacobsen KR, Kalliokoski O, Hansen AK, Hau J, Abelson KS (2012). The effect of automated blood sampling on corticosterone levels, body weight and daily food intake in permanently catheterized male BALB/c mice. In Vivo.

[95] Leuner B, Gould E (2010). Structural plasticity and hippocampal function. Annu Rev Psychol.

[96] Babu H, Cheung G, Kettenmann H, Palmer TD, Kempermann G (2007). Enriched monolayer precursor cell cultures from micro-dissected adult mouse dentate gyrus yield functional granule cell-like neurons. PLoS One.

[97] Walker TL, Kempermann G One mouse, two cultures: isolation and culture of adult neural stem cells from the two neurogenic zones of individual mice. J Vis Exp. 2014;84:e51225. 10.3791/51225 PMC4131911 24637893

[98] Crudo A, Suderman M, Moisiadis VG, Petropoulos S, Kostaki A, Hallett M (2013). Glucocorticoid programming of the fetal male hippocampal epigenome. Endocrinology.

[99] Lie DC, Colamarino SA, Song HJ, Desire L, Mira H, Consiglio A (2005). Wnt signalling regulates adult hippocampal neurogenesis. Nature.

[100] Seib DR, Corsini NS, Ellwanger K, Plaas C, Mateos A, Pitzer C (2013). Loss of Dickkopf-1 restores neurogenesis in old age and counteracts cognitive decline. Cell Stem Cell.

[101] Wang Q, Liu Y, Zou X, Wang Q, An M, Guan X (2008). The hippocampal proteomic analysis of senescence-accelerated mouse: implications of Uchl3 and mitofilin in cognitive disorder and mitochondria dysfunction in SAMP8. Neurochem Res.

[102] Trinchero MF, Buttner KA, Sulkes Cuevas JN, Temprana SG, Fontanet PA, Monzon-Salinas MC (2017). High Plasticity of New Granule Cells in the Aging Hippocampus. Cell Rep.

[103] Bornstein SR, Steenblock C, Chrousos GP, Schally AV, Beuschlein F, Kline G et al. Stress-inducible-stem cells: a new view on endocrine, metabolic and mental disease? Mol Psychiatry. 2019;24:2–9. 10.1038/s41380-018-0244-9 PMC6755998 30242231

[104] Sapolsky RM (1996). Why stress is bad for your brain. Science.

[105] Sapolsky RM (1999). Glucocorticoids, stress, and their adverse neurological effects: relevance to aging. Exp Gerontol.

[106] Lupien SJ, de Leon M, de Santi S, Convit A, Tarshish C, Nair NP (1998). Cortisol levels during human aging predict hippocampal atrophy and memory deficits. Nat Neurosci.

[107] McEwen BS, Nasca C, Gray JD (2016). Stress effects on neuronal structure: hippocampus, amygdala, and prefrontal cortex. Neuropsychopharmacology.

[108] Sorrells SF, Paredes MF, Cebrian-Silla A, Sandoval K, Qi D, Kelley KW (2018). Human hippocampal neurogenesis drops sharply in children to undetectable levels in adults. Nature.

[109] Schloesser RJ, Lehmann M, Martinowich K, Manji HK, Herkenham M (2010). Environmental enrichment requires adult neurogenesis to facilitate the recovery from psychosocial stress. Mol Psychiatry.

[110] Freund J, Brandmaier AM, Lewejohann L, Kirste I, Kritzler M, Kruger A (2013). Emergence of individuality in genetically identical mice. Science.

[111] Lemaire V, Aurousseau C, Le Moal M, Abrous DN (1999). Behavioural trait of reactivity to novelty is related to hippocampal neurogenesis. Eur J Neurosci.

[112] Anacker C, Luna VM, Stevens GS, Millette A, Shores R, Jimenez JC (2018). Hippocampal neurogenesis confers stress resilience by inhibiting the ventral dentate gyrus. Nature.

[113] Mignone JL, Kukekov V, Chiang AS, Steindler D, Enikolopov G (2004). Neural stem and progenitor cells in nestin-GFP transgenic mice. J Comp Neurol.

[114] Flurkey K, Currer JM, Harrison DE The Mouse in Aging Research. The Jackson laboratory handbook on genetically standardized mice, 6th edition, vol. 6th edition. The Jackson Laboratory Press, Bar Harbor, USA; 2007.

[115] Fluttert M, Dalm S, Oitzl MS (2000). A refined method for sequential blood sampling by tail incision in rats. Lab Anim.

[116] Beining M, Jungenitz T, Radic T, Deller T, Cuntz H, Jedlicka P (2017). Adult-born dentate granule cells show a critical period of dendritic reorganization and are distinct from developmentally born cells. Brain Struct Funct.

[117] Langmead B, Trapnell C, Pop M, Salzberg SL (2009). Ultrafast and memory-efficient alignment of short DNA sequences to the human genome. Genome Biol.

[118] Schouten M, Fratantoni SA, Hubens CJ, Piersma SR, Pham TV, Bielefeld P (2015). MicroRNA-124 and −137 cooperativity controls caspase-3 activity through BCL2L13 in hippocampal neural stem cells. Sci Rep.

[119] Zuberi K, Franz M, Rodriguez H, Montojo J, Lopes CT, Bader GD (2013). GeneMANIA prediction server 2013 update. Nucleic Acids Res.

[120] Faul F, Erdfelder E, Lang AG, Buchner A (2007). G*Power 3: a flexible statistical power analysis program for the social, behavioral, and biomedical sciences. Behav Res Methods.

