# Supplementary material for: Circadian glucocorticoid oscillations preserve a population of adult hippocampal neural stem cells in the aging brain
Source: Mol Psychiatry. 2019 Jun 20;25(7):1382–405. doi: 10.1038/s41380-019-0440-2 (PMC7303016; doi:10.1038/s41380-019-0440-2)
Supplement: Supplementary file 1 — Supplemental text [file 41380_2019_440_MOESM1_ESM.docx]

**Legends to Supplemental Figures and Tables**

**Figure S1, related to Figure 1 – NSPC subtypes decrease with age following different kinetics.**

Nestin-GFP (green) and PCNA (red) immunoreactivity in the DG of (A) 3 or (B) 18 month-old mice. (C) Representative confocal image showing examples of Nestin-GFP+/GFAP+ (Type-1 and Type-2a) and Nestin-GFP+/GFAP- (Type-2b) NSPC in the SGZ of the DG. The dashed white line shows a transversal cell section of each NSPC type. (D-F) Histogram of the transversal sections in C, showing fluorescent intensity signals for GFP (green), GFAP (red) and DNA (blue). Nestin-GFP (green) and GFAP (red) immunoreactivity in the DG of (G) 3; (H) 10; or (I) 18 month-old mice. Scale bars=50 (A and B), 10 (C) and 25 (G-I) µm. (J) Best-fit curves and 95% confidence intervals of GZ volume (squares) and total Nestin-GFP+ NSPC numbers (circles) per mm^3^ GZ. GZ volume was not significantly different across ages (*p*>0.05, vs. 3 month-old mice, one-way ANOVA). Nestin-GFP+ NSPC fitted to an exponential decay curve (*p*<0.05, *F*-test, calculated t_1/2_= 5.44 months as indicated in the figures). (K) Best-fit curves and 95% confidence intervals showing Type-1 (circles), Type-2a (triangles) and Type-2b (squares) NSPC numbers per mm^3^ GZ vs. age. Datapoints are mean±SEM (n=5, **p*<0.05, ***p*<0.01 and ****p*<0.001 vs. 3 month-old mice, one-way ANOVA) and NSPC population half lives (*t_1/2_*) are indicated in the figures. Type-1 and Type-2a NSPC fitted best to linear decay curves (*p* <0.05, *F*-test; calculated t_1/2_=18.5 and 21.9 months respectively), while Type-2b NSPC fitted best to exponential decay (*p*<0.05, *F*-test, calculated t_1/2_=1.05 months). (L) Relative numbers of GR^+^ (full bars, full circles) or GR- (dashed bars, open circles) Type-1 (red), -2a (green) or -2b (blue) NSPC per DG. Bars are mean±SEM (n=5). Differences between GR^+^/GR^-^ populations (**p*<0.05, ****p*<0.001) and time-points (^#^*p*<0.05, ^##^*p*<0.01, ^###^*p*<0.001) vs. 3-month-old are indicated (one-way ANOVA).

**Figure S2, related to Figure 1 – Nestin, GR expression and decay kinetics of NSPC subpopulations.**

(A) Representative confocal image depicting the DG of 3 month-old Nestin-GFP mice stained for GFP (green), GFAP (blue) and Nestin (red). (A’) The dashed white line shows a transversal cell section of a Type-1 NSPC. (A”) Histogram of the transversal section in A’, showing fluorescent intensity signals for GFP, GFAP and Nestin. (B) Representative confocal image depicting the DG of 12 month-old Nestin-GFP mice stained for GFP (green), GFAP (blue) and Nestin (red). (B’) The dashed white line shows a transversal cell section of a Type-1 NSPC. (B”) Histogram of the transversal section in B’, showing fluorescent intensity signals for GFP, GFAP and Nestin. Scale bars in A and B = 24 µm and in A’ and B’=12 µm. (C) Type-1 Nestin^+^/GFAP^+^ NSPC Nestin-GFP signal intensity in 3 and 12 month-old animals is shown as red bars representing mean grey value ±SEM and open circles mean grey values per mouse (n=5, ns, 3 vs. 12 months, Student’s t-test). (D) GR intensities in non-cellular regions of the hilus used as background signal (BG; white bar and red circles) and in Type-1/GR^+^ (red solid bar and full circles), Type-1/GR^-^ (red dashed bar and open circles), Type-2a/GR^+^ (green solid bar and full circles), Type-2a/GR^-^ (green dashed bar and open circles), Type-2b/GR^+^ (blue solid bar and full circles) and Type-2b/GR^-^ (blue dashed bar and open circles) NSPC. Data are mean grey value ±SEM and open circles mean grey values of individual mice (n=5, ** *p*<0.001, one-way ANOVA). (E) 5-mC intensity in Type-1 NSPC present in 3 and 18 month-old animals and depicted as red bars showing mean grey value ±SEM (n=5, ****p*<0.001, 3 vs. 18 months, Student’s t-test). Best-fit comparison of cell numbers vs. age curves. (F) Type-1/GR^+^ cells; red line=exponential curve fitting, black dashed line=linear fitting. (G) Type-2a/GR^+^ cells; green line=exponential curve fitting, black dashed line=linear curve fitting. (H) Type-2b/GR^+^ cells; blue line=exponential curve fitting, black dashed line=linear curve fitting. (I) Type-1/GR^-^ cells; red line=exponential curve fitting, black dashed line=linear curve fitting. (J) Type-2a/GR^-^ cells; green line=exponential curve fitting, black dashed line=linear curve fitting. (K) Type-2b/GR^-^ cells; blue line=exponential curve fitting, black dashed line=linear curve fitting. Data point values (polygons in F-K) are expressed as mean±SEM (n=5 mice). Best-fit curve values are expressed as mean±95% confidence interval. Linear or exponential best curve fitting was tested using a sum of squares *F*-test *p* values are shown in the plots, best fit to an exponential model is indicated with a *p*<0.05 (I-K).

**Figure S3, related to Figure 1 - Linear decay of GR+ NSPC correlates with age-associated increases in circadian CORT amplitude.**

Plots displaying linear regression between the circadian amplitude of CORT oscillations and the number of total Type-1 (red line and full circles), GR^+^ Type-1 (orange line and open squares) and GR- Type-1 (pink line and open diamonds) cells (A) the number of total Type-2a (dark green line and full squares), GR^+^ Type-2a (lighter green line and open triangles) and GR^-^ Type-2a (lightest green line and asterisks) cells (B) and the number of total Type-2b (dark blue line and upward-pointing full triangles), GR^+^ Type-2b (lighter blue line and downward-pointing open triangles) and GR^-^ Type-2b (lightest blue line and stars) cells (C). All regressions shown in had a significant (*p*<0.05) Pearson correlation and *p-values* shown in the figure indicate significant (*p*<0.05) deviations from a slope=0.

**Figure S4, related to Figures 2-5 - Experimental design used to model circadian GC oscillations and experimental validation.** (See also Supplemental Experimental Procedures).

(A) Schematic of the experimental setup used to obtain data shown in Figure 2. (B) Schematic of the experimental setup used to obtain data shown in Figure 3A-E and genotyping results of GR^wt/wt^ (single 219 bp band) and GR^fl/wt^ (both a 219 and 290 bp band) used in these studies. (C) Representative Z-stacked confocal image of Nestin-GFP^+^ Type-1 NSPC in Nestin-GFP mice 3dpi of a non-targeting control siRNA (siNC), showing characteristic vertical process and triangular cell-body in the SGZ of the DG. (D) A magnified single Z-plane of the area boxed in C. The dashed white line shows a transversal cell section of a Type-1 NSPC. (D’) Histogram of the transversal section in D showing fluorescent intensity signal for GFP (green). (E) Representative Z-stacked confocal image of Nestin-GFP^+^ Type-1 NSPC in Nestin-GFP mice 3dpi of a siRNA targeting GFP (siGFP), showing characteristic vertical process and triangular cell-body in the SGZ of the DG. (F) A magnified single Z-plane of the area boxed in E. The dashed white line shows a transversal cell section of a Type-1 NSPC. (F’) Histogram of the transversal section in E, showing fluorescent intensity signal for GFP (green). (G) Quantification of GFP intensity in Type-1 cells 3 dpi of two siRNAs. Data are average GFP intensity (gray value) of individual mouse (open circles) ±SEM (n=3, ****p*<0.001, siNC vs. siGFP, Student’s t-test). Scale bars=12 µm (C-F). (H) Schematic of the experimental setup used to obtain the data shown in panels C-G and Figure 3E-H. Representative Z-stacked confocal images of Type-1 NSPC 1dpi of a negative control siRNA labeled with Cy3 (siNC^Cy3^). (I) a Nestin-GFP^+^/siNC^Cy3+^ cell with the characteristic vertical process and triangular cell-body of Type-1 cells in the SGZ. (I’) Higher magnification and split confocal channels and (K) orthogonal projection of the area boxed in I, showing DNA (blue), GFP (green) and Cy3 (red, indicated by arrow). The dashed white line in (J) shows a transversal cell section. (J’) Histogram of the transversal section in J, showing fluorescent intensity signal for DNA, GFP and siNC^Cy3^. Scale bars=12 µm (I-M). (L) Schematic of the experimental setup used to obtain data shown panels I-K. (M) Schematic of the experimental setup used to obtain data shown in Figure 5. (N) Schematic of the experimental setup used to obtain the *in vitro* data shown in Figures 4. (O) Plots displaying [CORT] levels in culture medium used for the experimental validation of the two CORT incubation regimens used *in vitro*.

**Figure S5, related to Figure 4 - NSPC cell cycle progression following distinct CORT concentrations and rhythms and their resulting total daily exposures.** (A) Schematic illustration of the different alternative CORT incubation regimen followed and (B) schematic representation of the resulting total daily CORT exposures (concentration*time exposed). (C) Overview on the effect of the incubation regimen depicted in (A-B) on cell cycle progression and (D) the individual cell cycle phases. Bars in (C-D) are average percentages of individual data-points (circles, % of total NSPC)±SEM, significant differences in (D) are indicated (n≥3, **p*<0.05, ***p*<0.01 and ****p*<0.001*,* one-way ANOVA with Tukey’s post hoc test). (E) Changes in SGK1 expression induced by the different CORT incubation regimen. Data are mean normalized fold change expression (relative to vehicle) of individual data-points (red circles)±SEM, significant differences are indicated (n≥4, **p*<0.05 and ****p*<0.001*,* one-way ANOVA with Tukey’s post hoc test). Ultr/circ: both ultradian and circadian oscillations applied.

**Figure S6, related to Figure 4 - Age-associated changes in 5-mC in vivo and changes in DNA methylation, DNMT expression and gene promoter methylation induced by oscillating CORT in NSPC cultures *in vitro*.** (A) Representative confocal Z-stacked images of Nestin-GFP (green), GFAP (red) and 5-mC (white) immunoreactivity in the GZ of 3 or 18-month-old mice. Arrowheads: Type-1 cells. Scale bars=20 µm (for quantifications see also Figure S2E). Changes in DNMT expression induced by oscillating or continuous CORT treatment for (B) 72h, (C) 72h followed by a 24h washout period (recovery) or (D) 72h followed by a 24h recovery followed by a 1x10-6 M CORT treatment (pulse) (further details in Figure S4 and Experimental Procedures). Data are mean normalized fold change (relative to vehicle) of individual data-points (red circles)±SEM (n≥3, *p<0.05, **p<0.01 and ***p<0.001 relative to vehicle; #p<0.05, ##p<0.01 and ###p<0.001, vs. oscillating CORT, one-way ANOVA with Tukey’s post hoc test). (E) Normalized global 5-mC levels after the treatments indicated in the graph legends. Data are mean normalized percentage of global DNA/promoter methylation (relative to vehicle) of individual data-points (open circles)±SEM, (n=6, **p<0.01 and ***p<0.001 relative to vehicle, one-way ANOVA with Tukey’s post hoc test). (F) Normalized gene promoter methylation after the treatments indicated in the legends. Data are percentages (bars and open circles) of normalized (relative to vehicle) read density levels at gene promoter regions (-2000 and +500bp of TSS. Further details in Experimental Procedures). GO analysis of (G) hyper-methylated or (H) hypo-methylated genes promoters (oscillating vs. continuous CORT; MBD2 read density difference ≥3). Bar graphs show number of annotated promoters per BP (red bars) and their hypergeometric FDR corrected p-values (blue bars). Further details in Table S1 and Figure S4N-O.

**Figure S7, related to Figure 4 - Oscillating CORT induces stable changes in gene promoter methylation in NSPC *in vitro*.** GO analysis of stably (A) hyper- or (C) hypo-methylated gene promoters (oscillating vs. continuous CORT after recovery; MBD2 read density difference ≥3). Bar graphs show number of annotated promoters per BP (red bars) and their hypergeometric FDR corrected p-values (blue bars). Pathway analysis of stably (B) hyper- or (D) hypo-methylated gene promoters (oscillating vs. continuous CORT after recovery; MBD2 read density difference ≥3). Embryonic organ morphogenesis (FDR corrected *p*=1.26*10-8), stem cell differentiation (FDR corrected *p*=4.51*10-5), cell fate commitment (FDR corrected *p*=6.39*10-4) and canonical Wnt signaling pathway (FDR corrected *p*=2.43*10-2) were significantly overrepresented networks.

**Figure S8, related to Figure 4 - CORT oscillation-induced stable alterations in gene promoter methylation *in vitro* and associated functional pathway analysis.**

(A) Pathway analysis of oscillating CORT-induced second-highest overrepresented BP of stable hyper-methylated promoters. GeneMANIA identified inorganic anion transport (red spheres; FDR corrected *p*=2.65*10^-10^) and positive regulation of glial cell differentiation (blue spheres; FDR corrected *p*=1.27*10^-2^) as significantly overrepresented functional networks. (B) Pathway analysis of oscillating CORT-induced third-highest overrepresented BP of stable hyper-methylated promoters. GeneMANIA identified negative regulation of cell activation (red spheres; FDR corrected *p*=6.25*10^-3^) and hindbrain development (blue spheres; FDR corrected *p*=6.25*10^-3^) as significantly overrepresented functional networks. (C) Pathway analysis of oscillating CORT-induced second-highest overrepresented BP of stable hypo-methylated promoters. GeneMANIA identified metal ion transmembrane transporter activity (orange spheres; FDR corrected *p*=1.67*10^-24^) and anion transmembrane transporter activity (blue spheres; FDR corrected *p*=1.17*10^-8^) as significantly overrepresented functional networks. (D) Pathway analysis of oscillating CORT-induced third-highest overrepresented BP of stable hypo-methylated promoters. GeneMANIA identified organic anion transport (blue spheres; FDR corrected *p*=2.63*10^-4^) and inorganic cation transmembrane transporter activity (red spheres; FDR corrected *p*=4.51*10^-19^) as significantly overrepresented functional networks.

**Figure S9. Highlights.**

1) NSPC populations expressing the GR predominate in the DG starting at middle age.

2) In older mice, in which the amplitude of GC oscillations is maximal, GR knockdown results in a strong activation of Type-1 cells, which is scarce in control animals of the same age.

3) In a mouse model of accelerated aging (SAMP8 mice), disruption of circadian GC oscillations results in lasting morphological changes in newborn granule neuron morphology, indicating alterations in their connectivity.

4) *In vitro* (not shown in the scheme), GC oscillations control cell cycle progression, DNMT expression and DNA methylation in specific gene promoters in NSPC. Although some of the changes in promoter methylation were transient, a large number were preserved in daughter NSPC, and affected, among others, genes involved in cell cycle control and in the canonical Wnt signalling pathway.

**Table S1, related to Figure 6 - Genecodis GO analysis of significantly overrepresented BP among gene promoters differentially methylated by oscillating CORT treatment *in vitro*.**

**Table S2, related to Figure 7 - Genecodis GO analysis of significantly overrepresented BP among gene promoters lastingly differentially methylated by oscillating CORT treatment *in vitro*.**
