# Supplementary figures and images for: Circadian glucocorticoid oscillations preserve a population of adult hippocampal neural stem cells in the aging brain
Source: Mol Psychiatry. 2019 Jun 20;25(7):1382–405. doi: 10.1038/s41380-019-0440-2 (PMC7303016; doi:10.1038/s41380-019-0440-2)

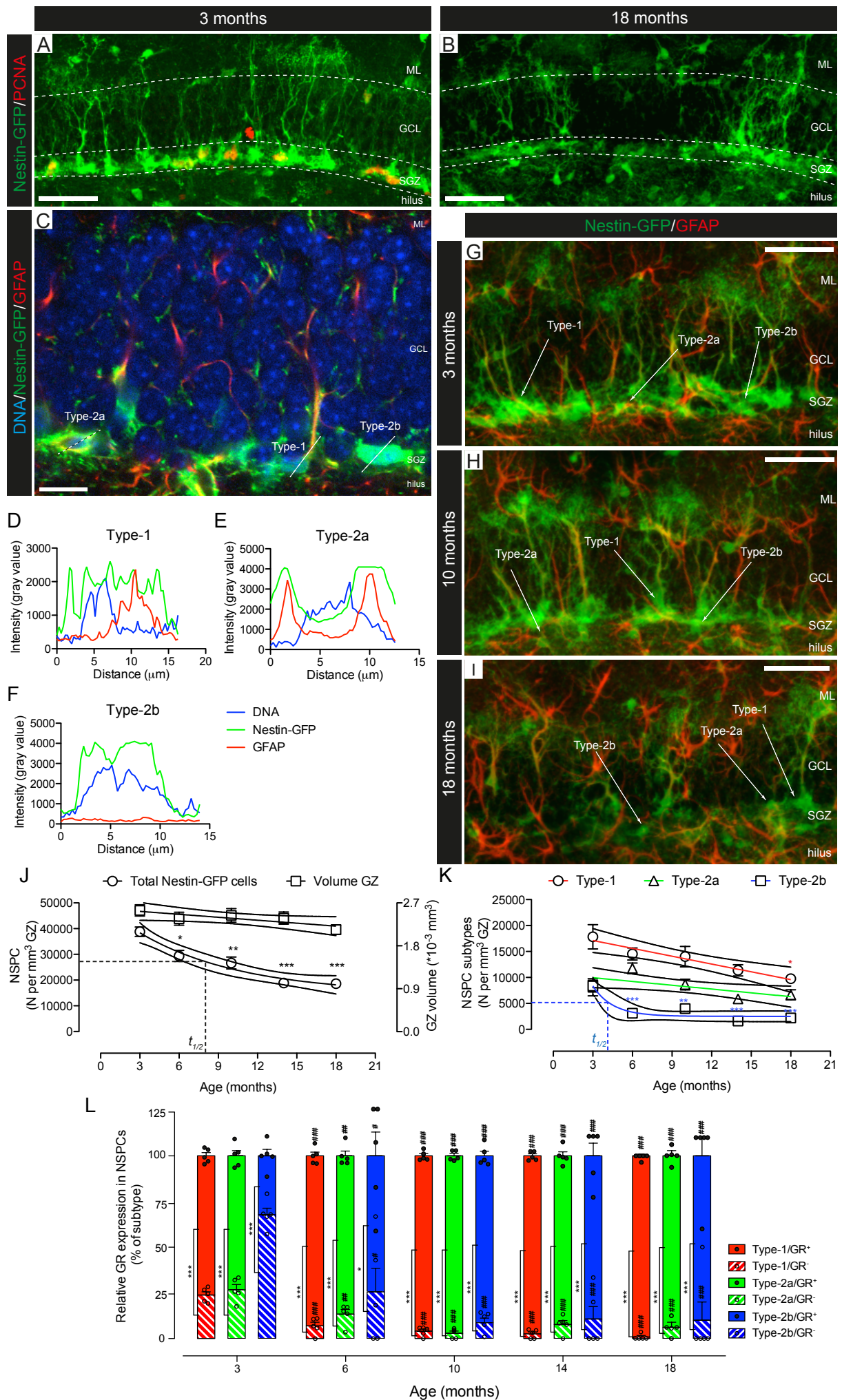

- Figure S1 Schouten *et al.* -

Supplement: Supplementary file 2 — Supplemental Figure 1 [file 41380_2019_440_MOESM2_ESM.pdf]

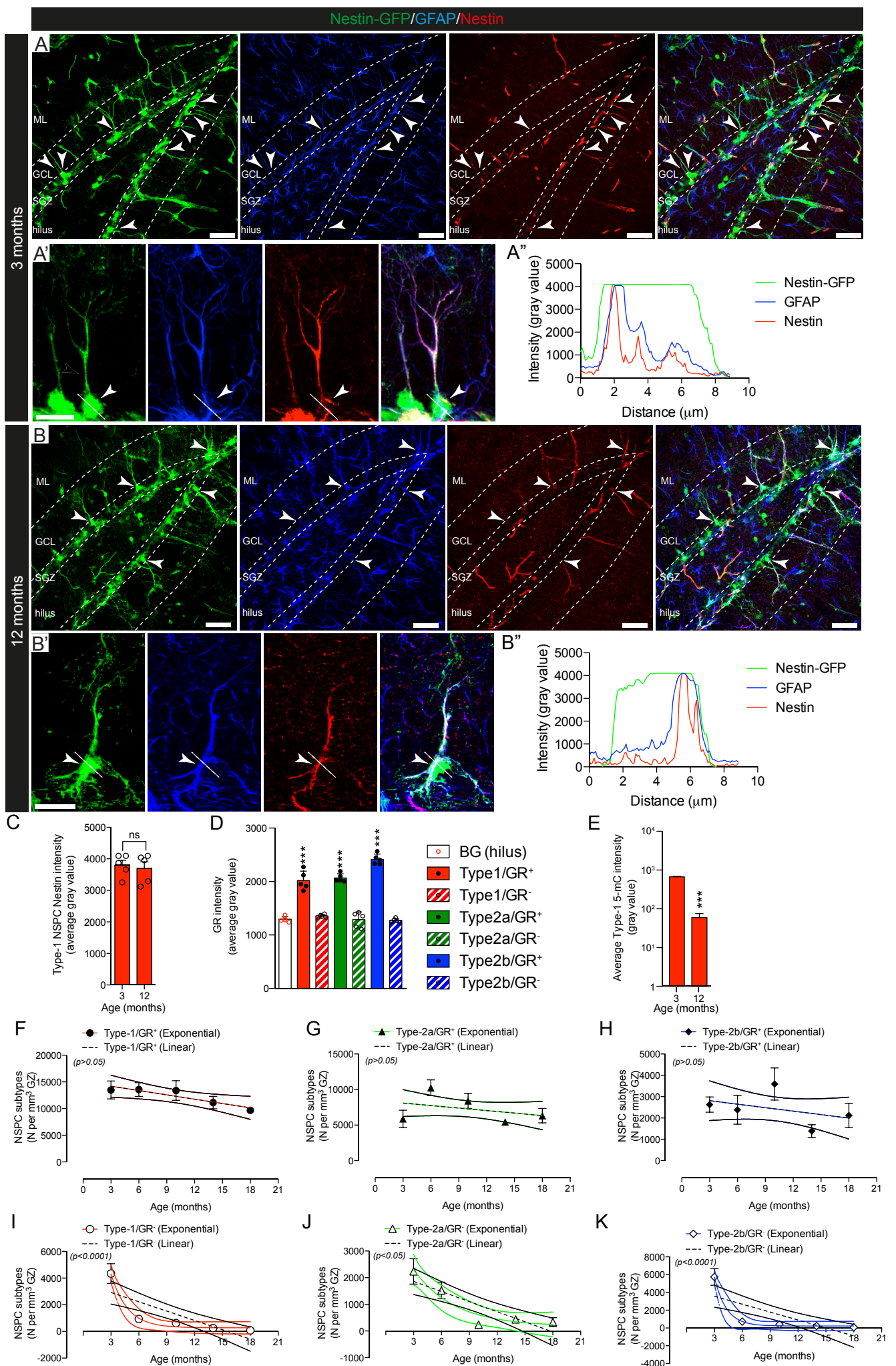

- Figure S2 Schouten *et al.* -

Supplement: Supplementary file 3 — Supplemental Figure 2 [file 41380_2019_440_MOESM3_ESM.pdf]

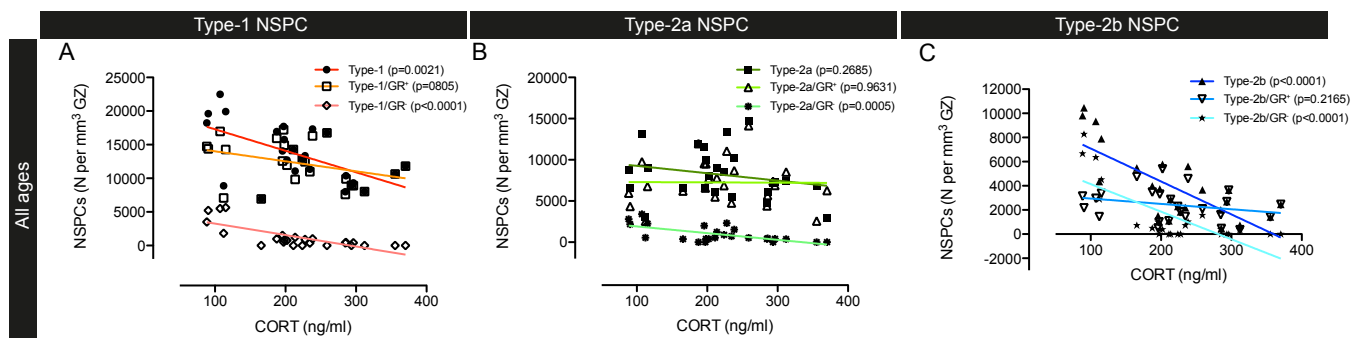

- Figure S3 Schouten *et al.* -

Supplement: Supplementary file 4 — Supplemental Figure 3 [file 41380_2019_440_MOESM4_ESM.pdf]

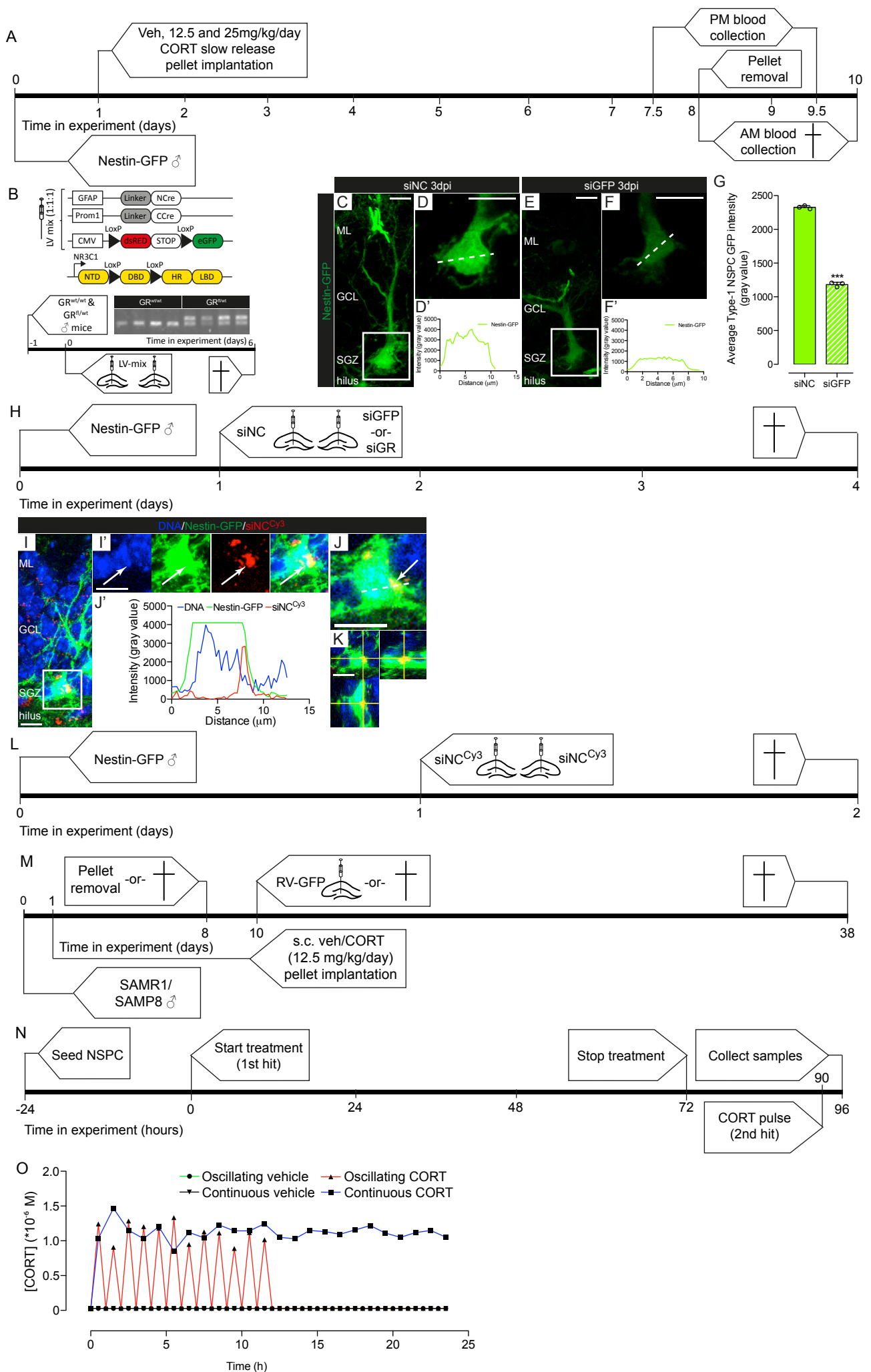

- Figure S4 Schouten *et al.* -

Supplement: Supplementary file 5 — Supplemental Figure 4 [file 41380_2019_440_MOESM5_ESM.pdf]

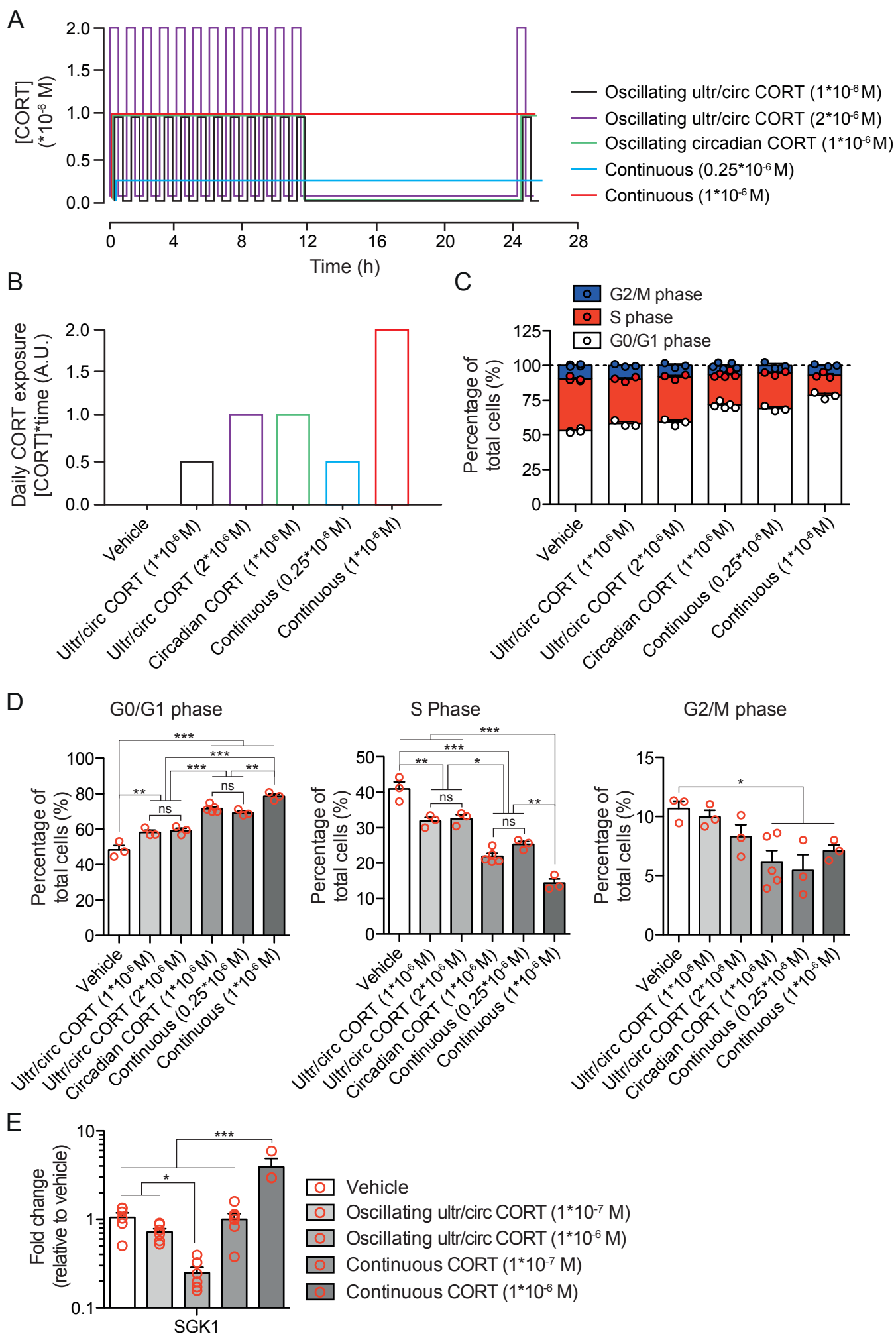

- Figure S5 Schouten *et al.* -

Supplement: Supplementary file 6 — Supplemental Figure 5 [file 41380_2019_440_MOESM6_ESM.pdf]

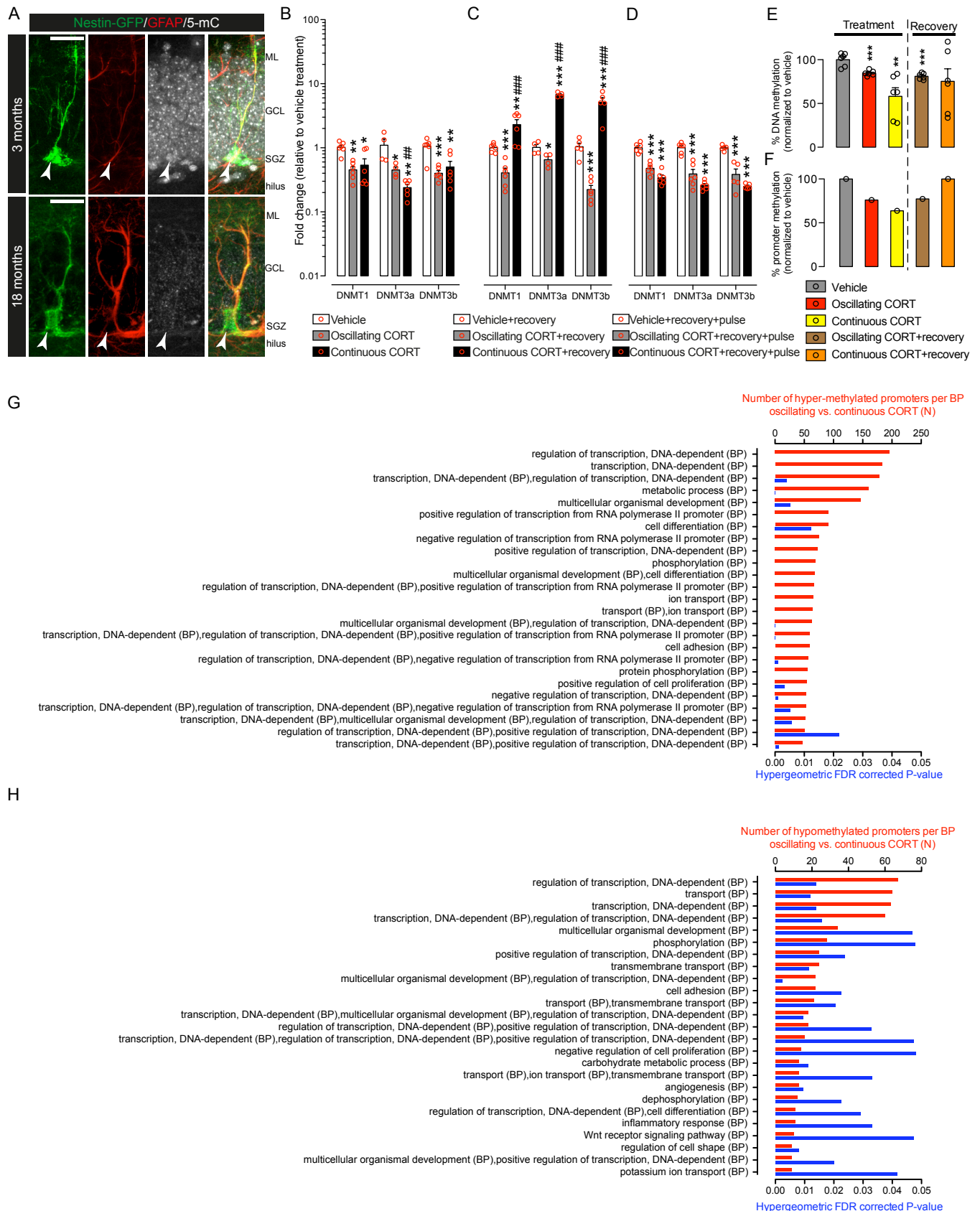

- Figure S6 Schouten *et al.* -

Supplement: Supplementary file 7 — Supplemental Figure 6 [file 41380_2019_440_MOESM7_ESM.pdf]

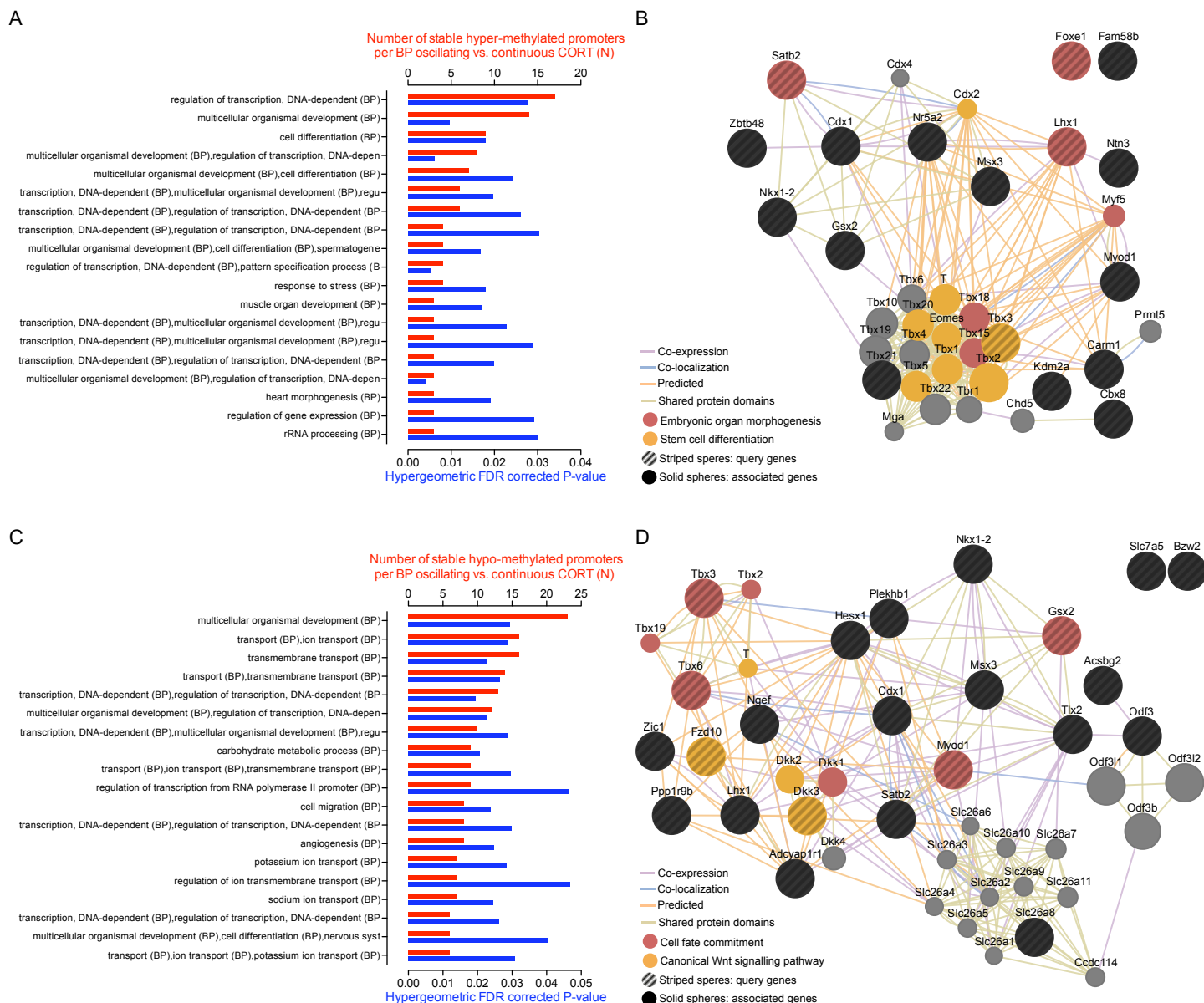

- Figure S7 Schouten *et al.* -

Supplement: Supplementary file 8 — Supplemental Figure 7 [file 41380_2019_440_MOESM8_ESM.pdf]

A

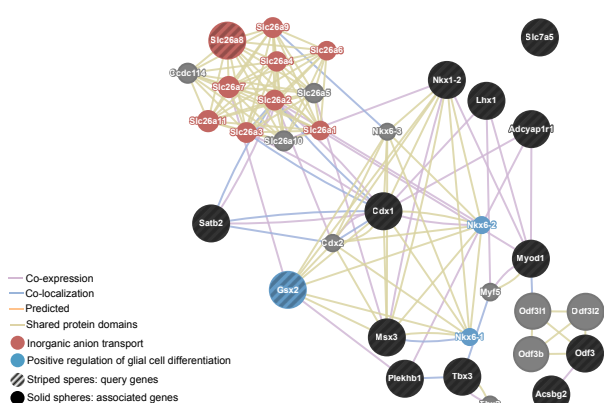

C

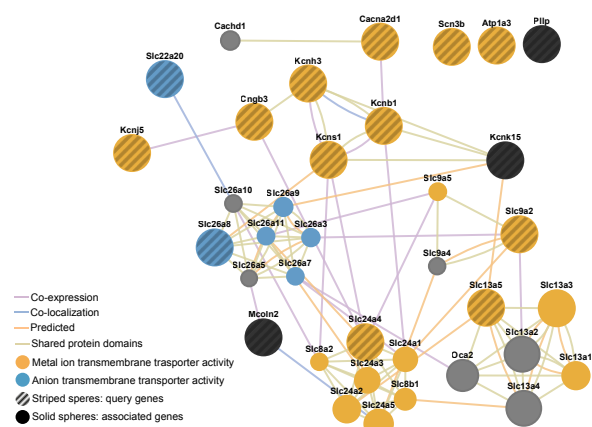

B

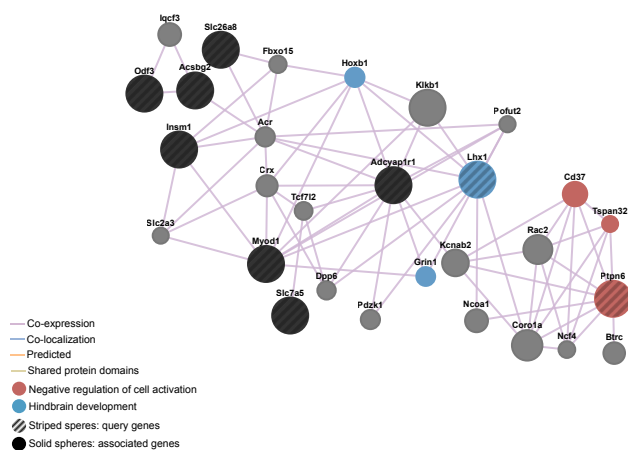

D

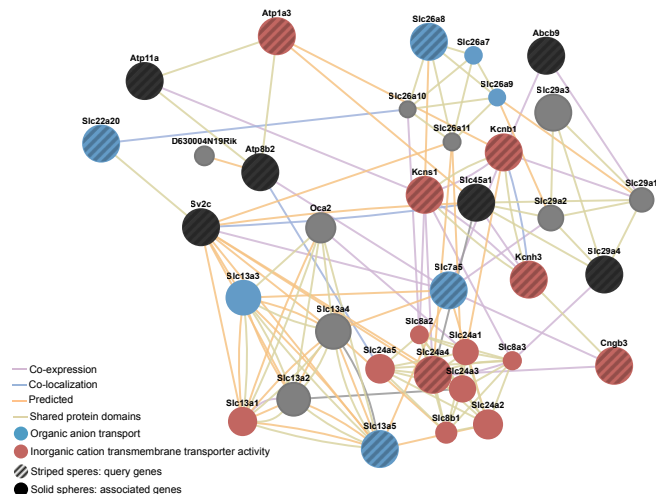

Supplement: Supplementary file 9 — Supplemental Figure 8 [file 41380_2019_440_MOESM9_ESM.pdf]

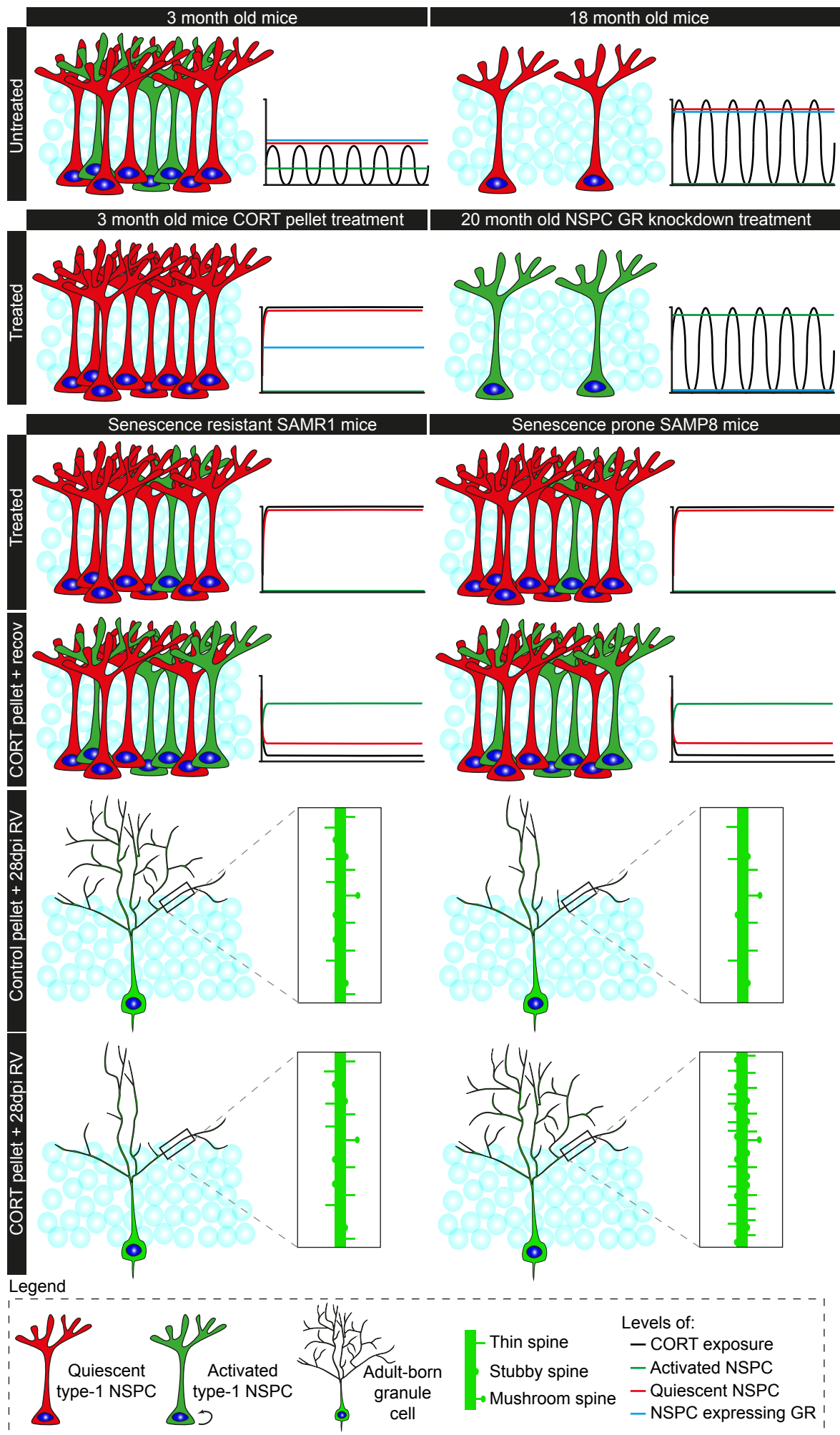

Supplement: Supplementary file 10 — Supplemental Figure 9 [file 41380_2019_440_MOESM10_ESM.pdf]
